# Severely ill and high-risk COVID-19 patients exhibit increased peripheral circulation of CD62L+ and perforin+ T cells

**DOI:** 10.3389/fimmu.2023.1113932

**Published:** 2023-02-02

**Authors:** Kelsey E. Lesteberg, Paula Araya, Katherine A. Waugh, Lakshmi Chauhan, Joaquin M. Espinosa, J. David Beckham

**Affiliations:** ^1^ Department of Medicine, Division of Infectious Diseases, University of Colorado School of Medicine, Aurora, CO, United States; ^2^ Department of Immunology and Microbiology, University of Colorado School of Medicine, Aurora, CO, United States; ^3^ Linda Crnic Institute for Down Syndrome, University of Colorado School of Medicine, Aurora, CO, United States; ^4^ Department of Pharmacology, University of Colorado School of Medicine, Aurora, CO, United States; ^5^ Department of Neurology, University of Colorado School of Medicine, Aurora, CO, United States; ^6^ Department of Medicine, Rocky Mountain VA Medical Center, Aurora, CO, United States

**Keywords:** COVID-19, T cells, CD62L, perforin, diabetes, hypertension

## Abstract

**Introduction:**

The emergence of SARS-CoV-2, which causes COVID-19, has led to over 400 million reported cases worldwide. COVID-19 disease ranges from asymptomatic infection to severe disease and may be impacted by individual immune differences.

**Methods:**

We used multiparameter flow cytometry to compare CD4+ and CD8+ T cell responses in severe (ICU admitted) and non-severe (admitted to observational unit) hospitalized COVID-19 patients.

**Results:**

We found that patients with severe COVID- 19 had greater frequencies of CD4+ T cells expressing CD62L compared to non-severe patients and greater frequencies of perforin+ CD8+ T cells compared to recovered patients. Furthermore, greater frequencies of CD62L+ CD4+ and CD8+ T cells were seen in severely ill diabetic patients compared to non-severe and non-diabetic patients, and increased CD62L+ CD4+ T cells were also seen in severely ill patients with hypertension.

**Discussion:**

This is the first report to show that CD62L+ T cells and perforin+ T cells are associated with severe COVID-19 illness and are significantly increased in patients with high-risk pre-existing conditions including older age and diabetes. These data provide a potential biological marker for severe COVID-19.

## Introduction

The coronavirus disease 2019 (COVID-19) pandemic, caused by severe acute respiratory syndrome coronavirus 2 (SARS-CoV-2), began in late 2019 and has caused more than 400 million reported infections worldwide (1, 2). COVID-19 disease spans a broad clinical spectrum, ranging from asymptomatic infection to severe disease requiring intensive care, mechanical ventilation, and prolonged medical support for survival. Common symptoms include fever, cough, shortness of breath, myalgia, and fatigue (3–5), and severe disease occurs in roughly 20% of patients (6). Advanced age and several chronic illnesses, including diabetes, cardiac disease, and lung disease, have been found to confer increased susceptibility to severe COVID-19 (7–9). Individuals who are immunocompromised, including those who have undergone solid organ transplantation or stem cell transplantation, are also at greater risk of severe disease (6, 10–12).

The broad clinical spectrum of COVID-19 suggests that differences in the immune systems of individual patients may be at play (13). Several studies have confirmed the presence of SARS-CoV-2 reactive CD4+ and CD8+ T cells in hospitalized patients (14–16). However, it is unclear if specific T-cell subsets are responsible for differences in disease severity, especially in high-risk individuals. T cells are crucial for eliminating viral infections, including respiratory infections due to SARS-CoV-2 and other coronaviruses (17–19). CD4+ T cells provide critical help to other immune cells, including necessary signals for B cell antibody production, and perform effector functions including cytokine secretion (20, 21). Cytotoxic CD8+ T cells mediate viral clearance by secreting the pore-forming protein perforin and apoptosis-inducing granzymes, which facilitate the death of infected cells (22, 23). Multiple studies have shown that SARS-CoV-2 infection leads to the activation of CD4+ T helper (Th) cells and cytotoxic CD8+ and CD4+ T cells, which tend to be of higher frequency in patients with more severe disease (13, 15, 24–29). While T cells are necessary for viral clearance, infiltration of adaptive immune cells into the lungs also results in increased inflammation and pulmonary edema, which may lead to lung injury (30). The immune system employs several mechanisms to avoid hyperactivation of the T cell response and therefore subsequent tissue damage, including the formation of regulatory CD4+ and CD8+ T cells (Tregs) and the binding of immune checkpoint and inhibitory receptors on T cells, such as PD-1, CTLA-4, and Tim-3, with their ligands (31–34). Although Tregs have been shown to limit acute lung injury and virus-mediated lung pathology, their role in COVID-19 pathology remains unclear (35–37). However, studies have demonstrated that COVID-19 patients have virus-specific T cells expressing PD-1 and Tim-3 that tend to increase with disease progression and severity (27, 38–42).

While recent studies have provided evidence of T cell subsets that may be associated with severe COVID-19, the mechanism by which T cells enter the lungs during SARS-CoV-2 infection is unclear. CD62L is a homing ligand which plays multiple roles in T cell trafficking, the most well characterized of which is its role in T cell trafficking to the lymph nodes (43). However, more recent evidence shows that CD62L also mediates the entry of T cells into non-lymphoid tissues (44), including lung tissue during influenza infection in mice (45), suggesting that it may be an important mediator of T cell trafficking to the lungs during respiratory infections. Additionally, despite the known association between COVID-19 severity and several underlying conditions, it is unclear whether or how chronic illness impacts the presence of T cell subsets during severe COVID-19. Therefore, we used multiparameter flow cytometry to examine the expression of T cell subsets, including CD62L+ T cells expressing markers of activation, Tregs, and immune checkpoint markers, as well as cytotoxic CD8+ T cells in severe and non-severe hospitalized COVID-19 patients, including patients with chronic illnesses such as diabetes and hypertension.

## Results

### Patient demographics

To examine the association between T cell subsets and severe COVID-19, blood samples were obtained from 30 patients enrolled into a prospective COVID-19 convalescent plasma (CCP) clinical trial after hospitalization with COVID-19 (46). Blood was drawn pre-CCP infusion from 30 randomly selected subjects out of 542 enrolled patients. All samples were obtained in April and May of 2020, before the availability of COVID-19 vaccines and emergence of SARS-CoV-2 variants of concern. In this study, patients were categorized as having non-severe disease if they were admitted for observation but not admitted into the intensive care unit (ICU) (N=14), and patients admitted into the ICU were categorized as having severe disease (N=16). The majority of severe patients required mechanical ventilation (43.8%, p=0.01) or extracorporeal membrane oxygenation (ECMO, 31.3%, p=0.04), while most non-severe patients only required oxygen *via* nasal canula (78.6%, p<0.00). Patient demographics, clinical characteristics, and treatments given for COVID-19 are listed in **Table 1**. The severe and non-severe groups were similar in terms of age (p=0.55) and sex (p>0.99)—the non-severe group had an average age of 54 (range 25-87) and 64.3% male sex, and the severe group had an average age of 58 (range 26-91) and 68.8% male sex. The two groups were also similar in terms of race (p=0.38 for multiracial, p>0.99 for all other categories) and ethnicity (p>0.99 Hispanic), as well as in the frequency of pre-existing conditions including diabetes (p=0.72), hypertension (p=0.71), immunosuppression (p>0.99), and malignancy (p>0.99). Additionally, many patients in both the severe and non-severe groups had body mass indexes (BMI) >30 (p=0.18) and as high as 57.6, indicative of obesity. As expected, the severe group was hospitalized significantly longer, with an average stay of 25.8 (range of 5-46) days compared to 10.4 (range of 4-42) days for the non-severe group (p<0.01). More severe patients were given prophylactic anticoagulants (87.5% severe, 50.0% non-severe, p=0.05) and corticosteroids (31.3% severe, 0% non-severe, p=0.04) compared to the non-severe group, whereas non-severe patients were treated more often with remdesivir (35.7% non-severe, 0% severe, p=0.01). One severe patient was treated with Tocilizumab (p>0.99). Lastly, no mortality was seen in the non-severe group, whereas the severe group had a mortality rate of 31.3% (p=0.04).

**Table 1:** Patient demographics and COVID-19 treatments.

		Non-Severe COVID-19	Severe COVID-19	p value
N		14	16	
Age (Median, range)		54 (25-87)	58 (26-91)	0.55
Sex (%, N)		64.3% (9) M 35.7% (5) F	68.8% (11) M 31.3% (5) F	>0.99
Race (%, N)				
	White	35.7% (5)	37.5% (6)	>0.99
	African American	7.1% (1)	12.5% (2)	>0.99
	Asian	0% (0)	6.3% (1)	>0.99
	Native American	0% (0)	6.3% (1)	>0.99
	Multiple	28.6% (4)	12.5% (2)	0.38
	Unknown	28.6% (4)	25.0% (4)	>0.99
Ethnicity (%, N)	Hispanic	57.1% (8)	62.5% (10)	>0.99
Comorbidities (%, N)				
	BMI (Median, range, N)	35.3 (30-57.6) (n=7)	30.2 (21-42.8) (n=15)	0.18
	Diabetes	64.3% (9)	56.3% (9)	0.72
	Hypertension	28.3% (4)	37.5% (6)	0.71
	Malignancy	14.3% (2)	12.5% (2)	>0.99
	Immune Suppressed	0% (0)	6.3% (1)	>0.99
	Lung Disease	0% (0)	18.8% (3)	0.23
	HIV+	7.1% (1)	0% (0)	0.47
	Pregnancy	7.1% (1)	0% (0)	0.47
	# Of Comorbidities (Median, range)	1.3 (0-4)	1.5 (0-5)	0.64
Supplemental Oxygen Support (%, N)				
	Nasal Canula	78.6% (11)	0% (0)	<0.01
	Face Mask	7.1% (1)	0% (0)	0.47
	Humidified High Flow Nasal Canula	14.3% (2)	18.8% (3)	>0.99
	Non-rebreather Mask	0% (0)	6.3% (1)	>0.99
	Mechanical Ventilation	0% (0)	43.8% (7)	**0.01**
	ECMO	0% (0)	31.3% (5)	**0.04**
Length of Hospitalization (Median, range)		10.4 (4-42)	25.8 (5-46)	**<0.01**
ICU Admission (%, N)		0% (0)	100% (16)	**<0.01**
COVID19 Treatment (%, N)				
	Anticoagulation	50.0% (7) Enoxaparin (6) Heparin (1)	87.5% (14) Lovenox (3) Heparin (7) Warfarin (1) Enoxaparin (1) Angiomax (1)	0.05
	Corticosteroids	0% (0)	31.3% (5) Dexamethasone (4) Hydrocortisone (1)	**0.04**
	Remdesivir	35.7% (5)	0% (0)	**0.01**
	Monoclonal Antibody	0% (0)	6.25% (1) Tocilizumab (1)	>0.99
	Convalescent Plasma	100% (14)	100% (16)	>0.99
Inpatient Mortality (%, N)		0% (0)	31.3% (5)	**0.04**
Hospital Day of Blood Draw (Median, range)		5 (2-16)	10 (1-31)	0.78

p values were generated through T test or Fisher exact test comparing non-severe vs. severe patients. p values less than 0.05 are highlighted in bold font.

### Seroconversion in non-severe and severe COVID-19 patients

To determine whether the patients had mounted an immune response to COVID-19, we performed 2 separate ELISAs—one which measures anti-SARS-CoV-2 spike receptor binding domain (RBD) IgG antibodies (47) and another which measures anti-SARS-CoV-2 nucleocapsid (N) IgG (48). 100% of severely ill patients exhibited positive IgG antibody to RBD, and all but 2 patients exhibited positive IgG antibody for N (84.6% reactive). In the non-severe group, the majority of patients exhibited positive IgG antibody to RBD and N, although at lower percentages than the severe group (78.6% RBD reactive and 64.3% N reactive, **Table 2**). Four patients, including 2 severe and 2 non-severe patients, were positive for RBD antibody but not N antibody. Patients who were negative for both RBD and N antibodies (3 non-severe patients) were immunocompromised, including 1 patient with lymphoma and one with HIV. Additionally, one pregnant patient was negative for both RBD and N (**Table S1**). Of the 4 patients in this cohort with malignancies, 2 were positive for both RBD and N antibodies, 1 patient was positive for RBD but not N, and one patient was negative for both antibody responses. These results are consistent with previous observations that patients with certain underlying conditions which suppress the immune response may be at risk for non-seroconversion after SARS-CoV-2 infection (49, 50). As expected, 100% of the healthy controls were negative for antibodies to both SARS-CoV 2 N and RBD, while 4/5 (80%) of the recovered patients were positive for both (1 patient was negative for both RBD and N). Overall, these results suggest that the majority of our patient population mounted an adaptive immune response to SARS-CoV-2.

**Table 2:** SARS-CoV-2 RBD and N ELISA reactivity.

		Non-Severe COVID-19 (n=14)	Severe COVID-19 (n=13)	COVID Recovered (n=5)	Healthy controls (n=4)
	RBD Reactive	78.6% (11)	100% (13)	80.0% (4)	0% (0)
	N Reactive	64.3% (9)	84.6% (11)	80.0% (4)	0% (0)
RBD and N Reactive		64.3% (9/14)	84.6% (11/13)	80.0% (4/5)	0% (0/4)
Only RBD Reactive		14.3% (2/14)	15.4% (2/13)	0% (0/5)	0% (0/4)
RBD and N Non-reactive		21.4% (3/14)	0% (0/13)	20% (1/5)	100% (4/4)

### Severe COVID-19 patients have greater frequencies of CD4+ T cells expressing CD62L

**Figure 1:**
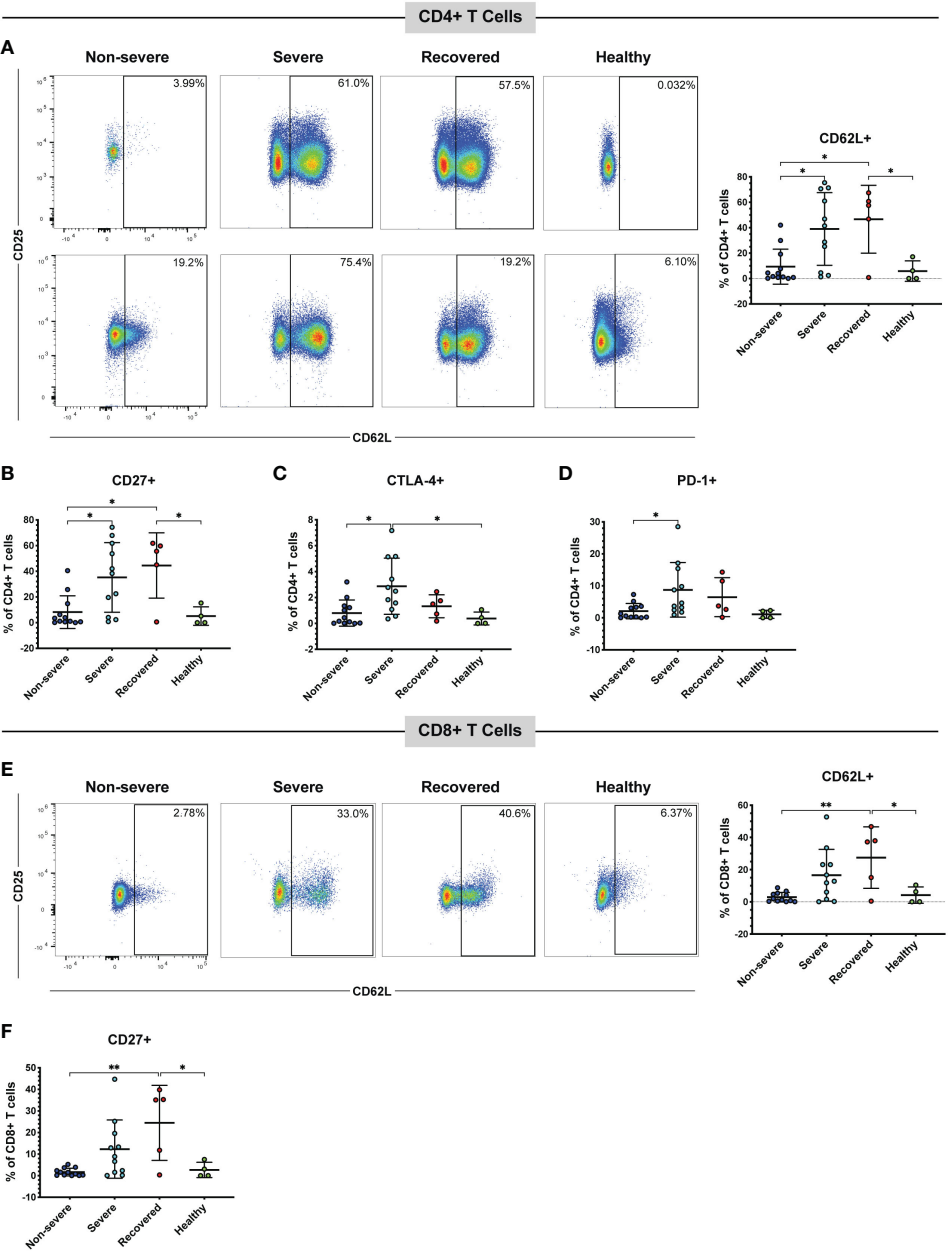
Severe and recovered COVID-19 patients have greater frequencies of CD62L+ T cells. The frequencies of CD4+ and CD8+ T cells (viable, CD14-, CD56-, CD19-, CD3+, CD4+ or CD8+ cells) expressing CD62L in non-severe and severe COVID-19 patients, recovered COVID-19 patients, and healthy controls were assessed by flow cytometry. **(A)** CD62L+ CD4+ T cells and representative flow cytometry plots. Each plot represents a different patient; percentages of CD4+ CD62L+ CD27+ cells **(B)**, CD4+ CD62L+ CTLA-4+ cells **(C)**, CD4+ CD62L+ PD-1+ cells **(D)**, CD8+ CD62L+ cells and representative flow plots **(E)**, and CD8+ CD62L+ CD27+ cells **(F)** within the total CD4+ T cell **(A-D)** and CD8+ T cell **(E, F)** populations; *p<0.05, **p<0.01 one-way ANOVA with multiple comparisons correction. Data are graphed as percentages of the total CD4+ or CD8+ T cell population.

CD62L, also called L-selectin, mediates the migration of lymphocytes to lymph nodes, the spleen, and virus-infected tissues (43, 51). Therefore, we measured the frequencies of CD62L+ CD4+ and CD8+ T cells in our patient cohort *via* flow cytometry. A detailed gating strategy for all flow cytometry analysis can be found in **Figure S1** Overall, the number of total T cells available for analysis from non-severe patients was often lower than that from severe patients. This is because non-severe patients tended to have a normal overall white blood cell count, whereas the severely ill patients tended to have a peripheral leukocytosis (data not shown). However, the percentages of total CD4+ (p=0.42) and CD8+ (p=0.48) T cells within the CD3+ population did not differ between the patient groups (**Figure S2A, B**). We found that severe COVID-19 patients had greater frequencies of CD4+ T cells expressing CD62L compared to non-severe COVID-19 patients (p=0.01), and recovered patients had greater frequencies of these cells compared to non-severe patients (p=0.02) and healthy controls (p=0.04) (**Figure 1A**). As these cells may home to lymph nodes or infected tissue, and severe COVID-19 is thought to be driven in part by an increase in activated T cells (28, 52–54), we examined the expression of the activation markers CD27 and CD25 on the CD62L+ T cells. The percentages of cells expressing CD27 (p=0.36) and CD25 (p=0.48) in the CD4+ CD62L+ population itself did not differ between the patient groups (**Figure S3A, B**). However, we also measured the frequencies of CD62L+ T cells co-expressing either CD27 or CD25 within the total CD4+ T cell population, as we wanted to understand whether the increase in CD62L+ cells was also driving an overall increase in activated cells. No significant differences were seen in the percentages of CD62L+ CD25+ cells (p=0.07, **Figure S4A**). However, severe COVID-19 patients had greater frequencies of CD27+ CD62L+ cells (**Figure 1B**, p=0.02) within the total CD4+ T cell population. Recovered patients also had greater CD4+ CD27+ CD62L+ cells compared to non-severe patients (p=0.01) and healthy controls (p=0.04) (**Figure 1B**).

Several studies have also confirmed the presence of immune checkpoint receptors, including CTLA-4, PD-1, and Tim-3 on the T cells of COVID-19 patients, often increasing as symptoms and disease severity progress (38–40, 42). Therefore, we measured the percentages of cells expressing CTLA-4, PD-1, or Tim-3 within the CD62L+ T cell populations (**Figure S3C-E**) and the percentages of CD62L+ cells also positive for each marker within the total CD4+ and CD8+ populations (**Figures 1C, D**, **Figure S4B**). CTLA-4 (p=0.72), Tim-3 (p=0.38), and PD-1 (p=0.32) percentages within the CD62L+ population did not differ between the patient groups (**Figure S3C-E**). However, severe COVID-19 patients had more CTLA-4+ CD62L+ cells (**Figure 1C**, p=0.01) and PD-1+ CD62L+ cells (**Figure 1D**, p=0.049) in the total CD4+ population compared to non-severe patients. Severe patients also had greater CTLA-4+ CD62L+ CD4+ T cells compared to healthy controls (p=0.04, **Figure 1C**). While a one-way ANOVA of Tim-3+ CD62L+ was significant (p=0.04), none of the multiple comparisons reached significance (**Figure S4B**).

Similar to the CD4+ T cells, recovered patients had greater CD62L+ CD8+ T cells compared to non-severe patients (p<0.01) and healthy controls (p=0.04). However, there was no statistically significant difference in CD62L+ CD8+ T cells between the severe and non-severe patients (p=0.06) despite a trend toward greater CD62L positivity in the severe patients (**Figure 1E**). There were no significant differences in the percentages of cells expressing CD27 (p=0.06), CD25 (p=0.77), CTLA-4 (p=0.21), Tim-3 (p=0.46), or PD-1 (p=0.20) in the CD8+ CD62L+ population (**Figure S3F-J**). However, within the total CD8+ T cell population, recovered patients had greater percentages of CD27+ CD62L+ cells compared to non-severe (p<0.01) and healthy controls (p=0.02). However, there was no significant difference between the non-severe and severe patients (p=0.10) (**Figure 1F**). There were no significant differences in CD25+ CD62L+ (p=0.07, **Figure S4C**), CTLA-4+ CD62L+ (p=0.07, **Figure S4D**), Tim-3+ CD62L+ (p=0.10, **Figure S4E**), or PD-1+ CD62L+ (p=0.02 one-way ANOVA, but no significant multiple comparisons, **Figure S4F**) CD8+ T cells. We next evaluated CD57 expression as a marker of cytotoxic potential in CD8+ T cells (55). While a one-way ANOVA analysis indicated a significant difference (p=0.02), none of the subsequent multiple comparisons were statistically significant (**Figure S4G**). These results suggest that severe COVID-19 patients have more CD62L+ T cells compared to non-severe COVID-19 patients. Although the expression of activation and immune checkpoint markers in the CD62L+ T cells is similar in the non-severe and severe patients (**Figure S3**), the increase in CD62L+ T cells seen in the severe group is also driving an increase in T cells positive for both CD62L and certain immune checkpoint or activation markers (**Figure 1**, **Figure S4**).

### Severe COVID-19 patients have greater frequencies of CD62L+ Tregs

**Figure 2:**
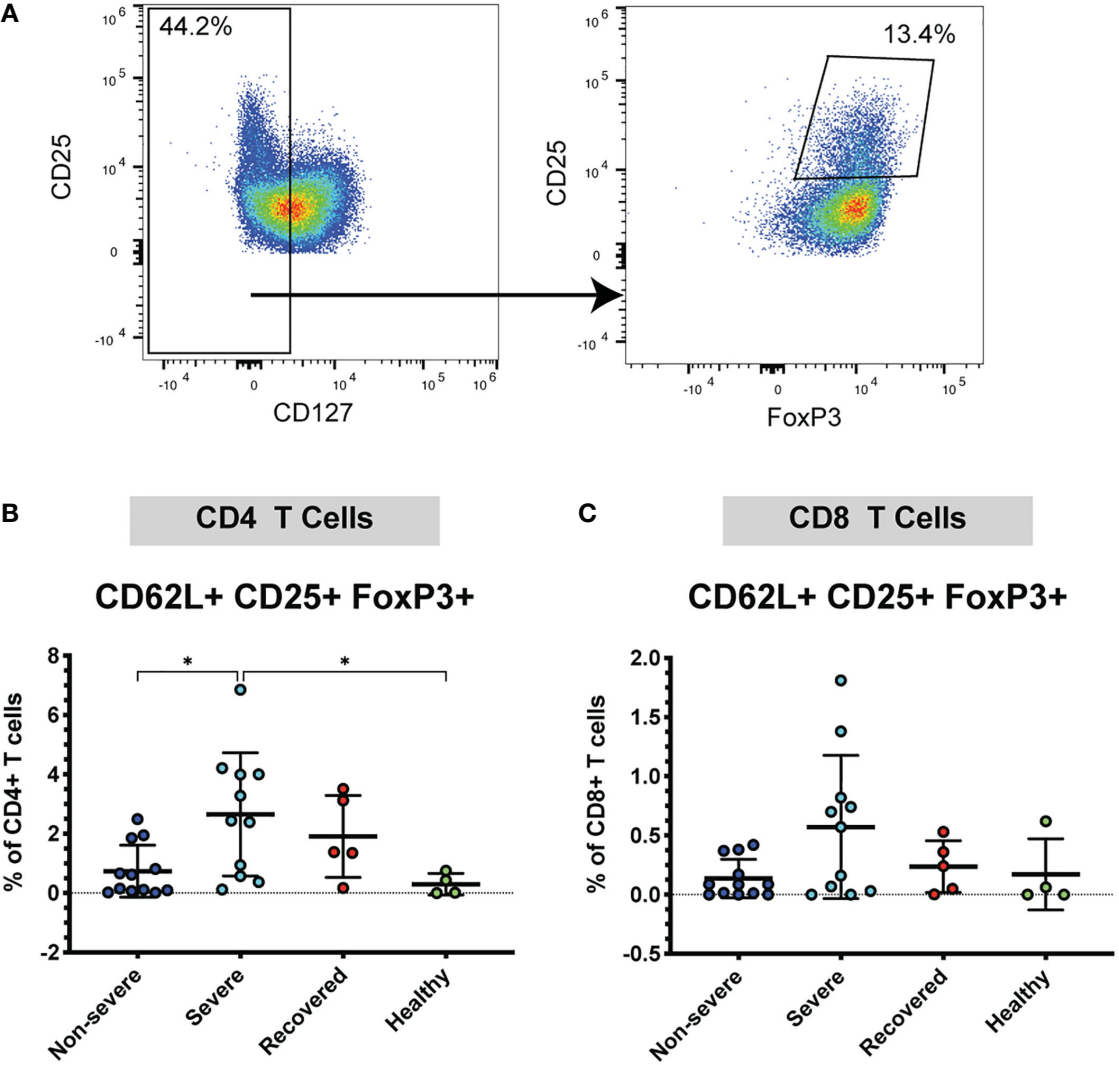
Severe COVID-19 patients have more CD4+ CD62L+ Tregs. The frequencies of CD4+ and CD8+ Tregs (viable, CD14- CD56-, CD19-, CD3+, CD4+ or CD8+, CD127lo, CD25+ FoxP3+) in non-severe and severe COVID-19 patients, recovered COVID-19 patients, and healthy controls were assessed by flow cytometry. **(A)** Example flow cytometry staining showing CD4+ CD62L+ T cells being gated on CD127lo, followed by gating for CD25+ FoxP3+ to identify Tregs. **(B)** CD4+ CD62L+ Tregs; **(C)** CD8+ CD62L+ Tregs. *p<0.05 one way ANOVA with multiple comparisons correction.

Given the increase in CD62L+ T cells in severe COVID-19 patients, we wanted to further understand whether these cells may be acting in a pro-inflammatory manner or may be acting to inhibit the immune response. Therefore, next examined the frequency CD62L+ Tregs, as Tregs are important immunomodulatory cells that may help limit lung pathology. Tregs were identified by low expression of CD127 and co-expression of CD25 and FoxP3 (CD127lo CD25+ FoxP3+) (**Figure 2A**). We found that severe patients had greater frequencies of CD62L+ CD4+ Tregs compared to non-severe (p=0.02) and healthy patients (p=0.04) (**Figure 2B**). No significant differences were seen in the CD8+ CD25+ FoxP3+ Tregs (p=0.07, **Figure 2C**). These results indicate that severe COVID-19 patients exhibit increased expression of CD4+ and CD8+ CD62L+ Tregs, which may form as a result of more severe pathology in this group. To determine whether the increase in CD62L+ Tregs was driving an overall increase in Tregs, we also measured the total frequencies of Tregs (regardless of CD62L expression) in the CD4+ and CD8+ populations. The frequencies of overall CD4+ (p=0.47) and CD8+ Tregs (p=0.13) did not differ significantly between the patient groups (**Figure S2C, D**).

### Recovered COVID-19 patients have increased frequencies of CD62L+ effector T cells

**Figure 3:**
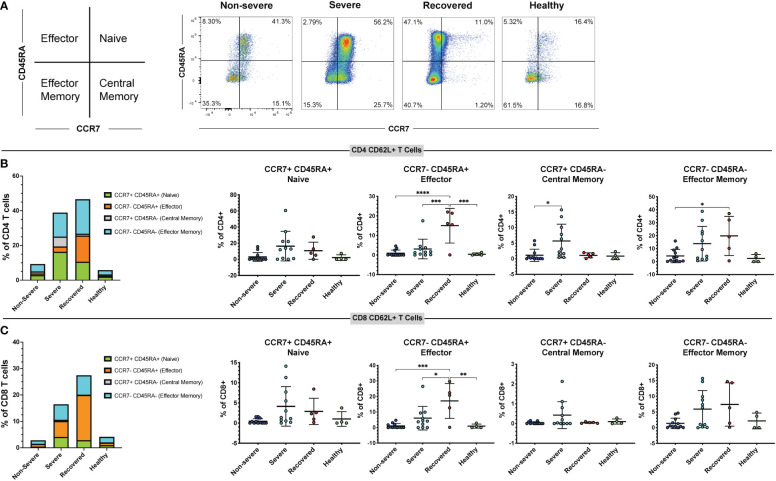
Immunological memory phenotyping of CD62L+ T cells. Expression of CCR7 and CD45RA on CD62L+ CD4+ and CD8+ T cells (viable, CD14-, CD56-, CD19-, CD3+, CD4+ or CD8+ cells) in non-severe and severe COVID-19 patients, recovered COVID-19 patients, and healthy controls was assessed by flow cytometry. **(A)** Representative diagram and flow cytometry plots showing naïve CD4+ CD62L+ T cells (CCR7+ CD45RA+), central memory CD4+ CD62L+ T cells (CCR7+ CD45RA-), effector memory CD4+ CD62L+ T cells (CCR7- CD45RA-), and effector CD4+ CD62L+ T cells (CCR7- CD45RA+); **(B)** percentages of CD62L+ naïve, effector, central memory, and effector memory cells within the CD4+ T cell population; **(C)** percentages of CD62L+ naïve, effector, central memory, and effector memory cells within the CD8+ T cell population. *p<0.05, **p<0.01, ***p<0.001 one-way ANOVA with multiple comparisons correction.

As CD62L expression differs on T cell subsets (56), we used the markers CCR7 and CD45RA to measure the frequencies of naïve (CCR7+ CD45RA+), effector (CCR7- CD45RA+), effector memory (CCR7- CD45RA-), and central memory (CCR7+ CD45RA-) T cells in the CD4+ CD62L+ and CD8+ CD62L+ populations (**Figure 3A**). Perhaps surprisingly, we found that the overall frequencies of naïve, central memory, effector, and effector memory CD4 and CD8 T cells did not differ significantly between our patient groups (**Figure S5**). However, when looking at the CD62L+ CD4+ T cell population, we found that recovered COVID-19 patients had greater frequencies of effector cells compared to non-severe patients (p<0.01), severe patients (p<0.01), and healthy controls (p<0.01). Additionally, severe COVID-19 patients had greater frequencies of central memory cells within the CD62L+ CD4+ T cells compared to recovered patients (p=0.03). No differences were seen in the frequencies of CD4+ CD62L+ effector memory (p=0.38) or naïve cells (p=0.50) (**Figure S6A-D**). In the CD8+ CD62L+ population, healthy controls had greater frequencies of central memory cells compared to non-severe COVID-19 patients (p=0.03), but no differences were seen in the naïve (p=0.32), effector (p=0.66), or effector memory cells (p=0.46) (**Figure S6E-H**). We next looked at the frequencies of naïve, central memory, effector, and effector memory cells expressing CD62L within the total CD4+ and CD8+ T cell populations. We found that severe patients had greater frequencies of central memory CD62L+ CD4+ T cells compared to non-severe patients (p=0.02), and recovered patients had more effector CD62L+ CD4+ T cells compared to non-severe patients (p<0.00), severe patients (p<0.01), and healthy controls (p<0.01) (**Figures 3A, B**). Additionally, recovered COVID-19 patients had higher frequencies of CD4+ CD62L+ effector memory cells compared to non-severe COVID-19 patients (p=0.04) (**Figure 3B**). No differences were seen in the CD4+ CD62L+ naïve cells (p=0.06, **Figure 3B**). In the CD8+ T cell population, recovered patients also had greater effector cell frequencies compared to non-severe patients (p<0.01), severe patients (p=0.01), and healthy controls (p<0.01) (**Figure 3C**). No differences were seen in the naïve (p=0.06) or central memory (p=0.13) CD8+ CD62L+ cells, and the effector memory cells were significant by one-way ANOVA (p=0.04), but the multiple comparisons were not significant (**Figure 3C**). These results show that recovery from COVID-19 results in increased CD62L+ effector CD4+ and CD8+ T cells.

### Severe COVID-19 patients have greater frequencies of perforin+ CD8+ T cells

**Figure 4:**
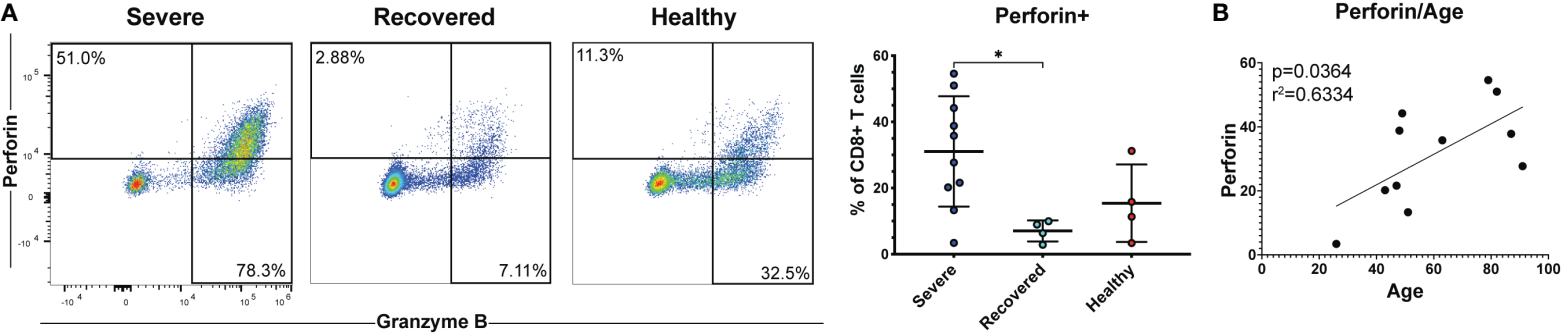
Severe COVID-19 patients have greater frequencies of perforin+ CD8+ T cells. **(A)** The frequencies of CD8+ T cells (viable, CD14-, CD56-, CD19-, CD3+, CD8+ cells) expressing perforin in non-severe and severe COVID-19 patients, recovered COVID-19 patients, and healthy controls were assessed by flow cytometry. **(B)** Correlation between age and perforin+ CD8+ T cells. *p<0.05 one-way ANOVA with multiple comparisons correction.

Cytotoxic CD8+ T cells secrete perforin and granzymes to kill virus-infected target cells and are crucial mediators of the anti-viral adaptive immune response (19, 22). Therefore, we measured the frequencies of CD8+ T cells expressing perforin and granzyme B as well as CD57, a marker of terminally differentiated cytotoxic T cells (55). Severe COVID-19 patients had greater percentages of perforin+ CD8+ T cells compared to recovered patients (**Figure 4A**, p=0.03). However, frequencies of granzyme B+ cells (**Figure S7A**, p=0.24) and CD57+ cells (**Figure S7B**, p=0.49) did not differ between the groups. Effector and cytotoxic T cell frequencies during viral infection tend to increase with age, and age is a known risk factor for COVID-19 severity (57, 58). Therefore, we examined whether perforin or granzyme B positivity correlated with age in our COVID-19 patients. We found that the frequency of perforin+ CD8+ T cells (**Figure 4B**, p=0.04, r=0.63), but not granzyme B+ CD8+ T cells (**Figure S7C**, p=0.25, r=0.38), was positively correlated with age. These results suggest that increased CD8+ T cell cytotoxicity associated with age may contribute to an increased risk of COVID-19 severity. However, we were unable to obtain this data in non-severe patients due to limited PBMCs in these samples.

### Severe COVID-19 patients with diabetes and hypertension have greater frequencies of T cells expressing CD62L

**Figure 5:**
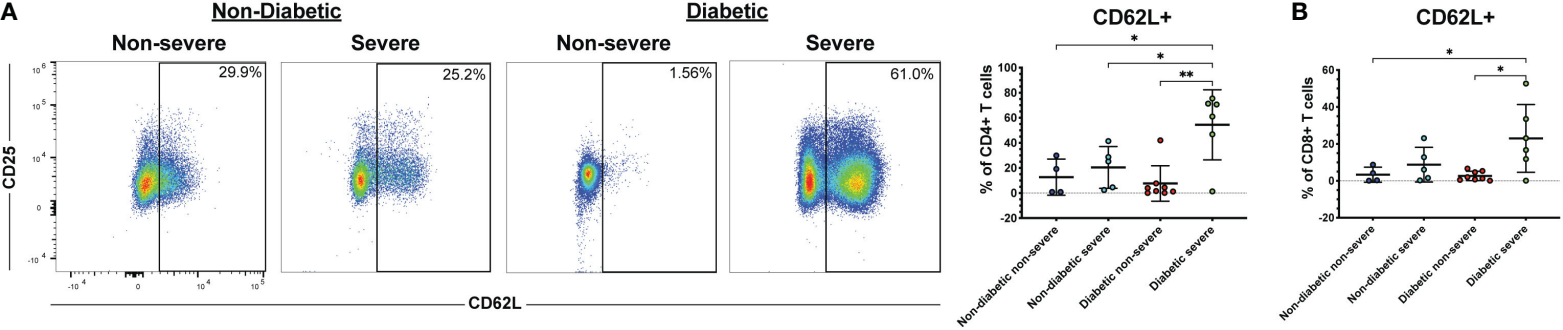
Diabetic severe COVID-19 patients have greater frequencies of CD62L+ T cells. The frequencies of CD4+ **(A)** and CD8+ T cells (viable, CD14-, CD56-, CD19-, CD3+, CD4+ or CD8+ cells) **(B)** expressing CD62L in severe and non-severe COVID-19 patients with or without diabetes were assessed by flow cytometry. *p<0.05, **p<0.01 one-way ANOVA with multiple comparisons correction.

**Figure 6:**
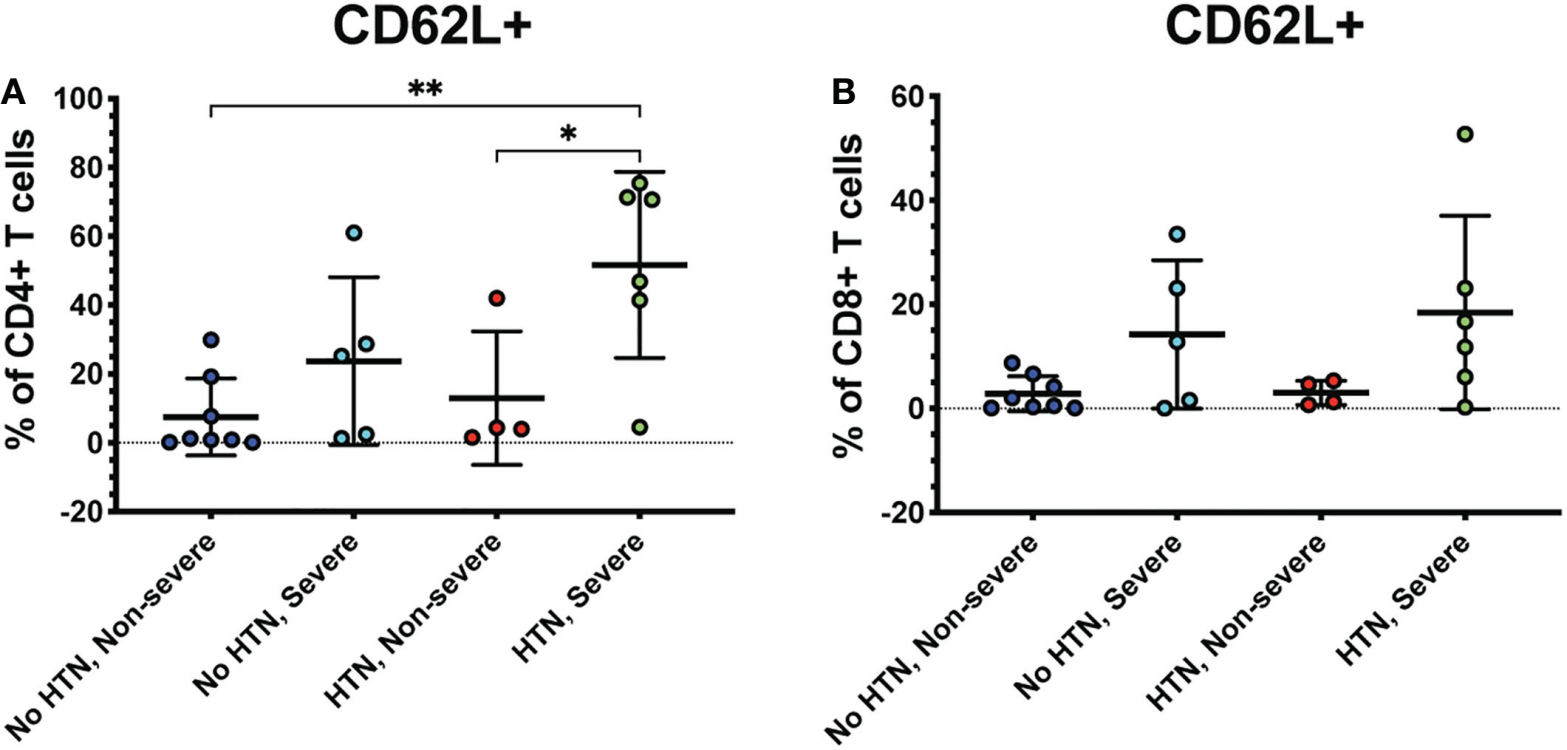
Severe COVID-19 patients with hypertension have greater frequencies of CD62L+ CD4+ T cells. The frequencies of CD4+ **(A)** and CD8+ T cells (viable, CD14-, CD56-, CD19-, CD3+, CD4+ or CD8+ cells) **(B)** expressing CD62L in severe and non-severe COVID-19 patients with or without hypertension were assessed by flow cytometry. *p<0.05, **p<0.01 one-way ANOVA with multiple comparisons correction.

Several co-morbidities, including diabetes and hypertension, confer greater risk of COVID-19 severity (7). Therefore, we compared severely ill and non-severely ill COVID-19 patients with or without diabetes or hypertension to determine whether the immune responses to COVID-19 differ in these patients. We found that patients with severe COVID-19 and diabetes had greater frequencies of CD62L+ CD4+ T cells compared to diabetics with non-severe COVID-19 (p<0.01) and non-diabetics with non-severe (p=0.02) or severe disease (p=0.04) (**Figure 5A**). Diabetic severely ill patients also had greater frequencies of CD8+ CD62L+ cells compared to diabetic non-severely ill patients (p=0.01) and non-severe patients without diabetes (p=0.04) (**Figure 5B**). Next, we made similar comparisons in patients with hypertension. Severely ill patients with hypertension had greater frequencies of CD4+ CD62L+ T cells compared to non-severely ill patients with hypertension (p=0.04) and non-severe patients without hypertension (p<0.01) (**Figure 6A**). However, no significant differences were seen in the CD62L+ CD8+ T cells in hypertensive patients (p=0.08) (**Figure 6B**). These results raise the possibility that increased CD62L+ T cell frequencies in patients with certain chronic illnesses may play a role in increased susceptibility to viral disease and highlight the need for further studies in these patient groups.

## Discussion

Several risk factors for severe COVID-19 disease have been identified, including advanced age, male sex, and multiple chronic illnesses including diabetes, hypertension, cardiovascular disease, lung disease, and immunodeficiencies (59, 60). However, the biological mechanisms underlying these susceptibilities are unclear. Here, we show for the first time that CD62L+ CD4+ T cells are increased in ICU patients with severe COVID-19 compared to non-severe hospitalized COVID-19 patients. Additionally, both CD4+ CD62L+ and CD8+ CD62L+ T cells were increased in severe COVID-19 patients with diabetes in our cohort, and CD4+ CD62L+ T cells were increased in severely ill COVID-19 patients with hypertension. While it is possible that the increase in CD62L+ T cells is a consequence of certain pre-existing illnesses, such as diabetes, further studies in these patients and in animal models are needed to determine a causative link. It is likewise possible that the increase in CD62L+ T cells in diabetic and hypertensive patients is driven by severe COVID-19 illness itself rather than any pre-existing conditions. There is also a possibility that the increase in CD62L+ T cells could be due to an increased average day of blood draw in the severe COVID-19 group. We think this is unlikely, as blood draws were obtained during a wide range of hospital days in both groups. Statistical analysis also indicated that there was no significant difference in blood draw date between the non-severe and severe groups (**Table 1**). Additionally, the increased average blood draw day in the severe COVID-19 group is impacted by both the increased length of hospital admission and the time it takes for severe COVID-19 symptoms to develop. It is also possible that age may impact the expression of CD62L, as has been previously shown (61). However, both our non-severe and severe COVID-19 groups had similar average ages and ranges of age. Additionally, much of our patient cohort had BMI >30, which classifies them as obese, and many studies have reported an increased risk of severe COVID-19 in obese individuals (62–64). While obesity has been shown to modify T cell phenotypes and expansion during viral infection (65, 66), it is difficult to tease out the difference between the effects of obesity and common co-morbidities, such as heart disease and diabetes, in humans.

Type 2 diabetes and hypertension are estimated to affect 462 million and 1.39 billion individuals worldwide, respectively (67, 68). The mechanisms underlying the increased susceptibility to COVID-19 in these patients are unclear but may include increased viral replication due to uncontrolled glucose levels, increased levels of inflammatory cytokines and reactive oxygen species, failure of insulin-responsive organs including the liver, and dysregulation of T and NK cell responses in diabetic patients (69). Patients with type 2 diabetes are also susceptible to severe disease outcomes with other viral infections, including West Nile virus, influenza, and other lower respiratory tract infections (70–73). Therefore, understanding the immune response to viral infections in diabetic patients is critical to protect this vulnerable patient population. Multiple studies in mice support a role for CD62L in the development of diabetes. For example, antibody-mediated blockade of CD62L in non-obese diabetic (NOD) mice protects against insulitis and diabetes (74, 75), particularly in neonatal mice. However, follow-up studies with CD62L^-/-^ NOD mice did not reveal any difference in diabetes induction between CD62L^+/+^ and CD62L^-/-^ mice (76), suggesting that other mechanisms may be able to compensate for the lack of CD62L during diabetes development. Further studies examining the role of CD62L in diabetes, and studies examining the immune response to COVID-19 in diabetic mouse models will likely provide important insight.

CD62L is expressed on naïve and central memory T cells, enabling their trafficking into peripheral lymph nodes (77, 78). CD62L has not traditionally been considered important for effector T cell homing, since it is downregulated following T cell priming (79). However, other studies have shown that CD62L is re-expressed on CD8+ T cells re-entering the bloodstream after activation in the lymph node and that it is necessary for the recruitment of CD8+ T cells to virus-infected tissues, including lung tissue following influenza infection (45). Future studies should examine whether CD62L+ T cells are similarly capable of homing to lung tissue during COVID-19 infection and whether they contribute to pulmonary disease pathology. Other studies have identified CXCR3, CXCR6, and CCR5 as other potential mediators of immune cell trafficking to the lungs during COVID-19 infection (80, 81), and one of these studies also demonstrated that T and NK cells that expressed these receptors in COVID-19 patients showed greater signs of activation (80). Further studies examining the contribution of these and other homing receptors in severe COVID-19 should provide critical information regarding the pathogenesis of COVID-19 and could perhaps explain differences in disease severity among individuals. While our study focused on human T cells in circulation, future studies examining the T cell phenotypes present in the lungs, as well as secondary lymphoid tissues, during COVID-19 in animal models are important and should also provide important insight into tissue-specific functions of T cells during viral infections.

In our study, severely ill COVID-19 patients had greater frequencies of CD4+ T cells co-expressing CD62L and immune checkpoint markers, specifically PD-1 and CTLA-4. T cells are critically important for mediating clearance of viral infections, including several respiratory infections. However, this process can also lead to organ damage and progression of other disease pathology if these responses are left unchecked. Immune checkpoint molecules and inhibitory receptors, such as PD-1, CTLA-4, and Tim-3, act as brakes for the immune response (31–33, 82). PD-1 is expressed on T cells in acute viral infections, including HBV and HCV, where it may play a role in dampening the T cell response during the later stages of infection to prevent organ damage (83, 84). In COVID-19 patients, one study demonstrated that PD-1 and Tim-3 were increased in expression as COVID-19 symptoms progressed while another found increased PD-1 expression in patients with active disease compared to patients who had recovered from mild disease (27, 39). Although PD-1 and other immune checkpoint receptors are often considered markers of exhausted cells, multiple lines of evidence suggest that PD-1+ T cells maintain at least some effector functions. In COVID-19 patients, PD-1+ CD8+ T cells have been found to express perforin and granzyme, proliferate upon re-stimulation, and express interferon gamma (38, 41, 85). Therefore, it cannot be assumed that T cells expressing immune checkpoint receptors in COVID patients are non-functional. The exact contribution of PD-1 in either promoting or preventing COVID-19 pathology needs further study. We also found higher frequencies of CD62L+ CD4+ and CD62L+ CD8+ Tregs in our severe COVID-19 patients. Previous studies have reported that CD62L+ Tregs have superior suppressive functions and proliferative capacity compared to CD62L- T cells (86, 87). However, the exact role of Tregs in COVID-19 has not yet been elucidated. It has been reported that patients with prolonged SARS-CoV-2 positivity have greater frequencies of Tregs (88), which could suggest that the persistence of Tregs in COVID-19 may prevent viral clearance. Future studies should focus on defining the roles of Tregs and immune checkpoints in COVID-19, especially focusing on T cells with potential tissue homing capabilities and defining the suppressive capabilities of CD62L+ Tregs in COVID-19.

We also found that severely ill COVID-19 patients have greater frequencies of perforin+ CD8+ T cells compared to recovered patients, indicating greater cytotoxic potential in these patients. In particular, the frequency of perforin+ CD8+ T cells in severe COVID-19 patients positively correlated with age. Advanced age is known to confer a greater risk of severe COVID-19, as well as susceptibility to other viral infections (89, 90). Aging results in numerous changes to the immune system, a process termed immunosenescence. A major feature of immunosenescence is a shift towards highly differentiated T cells and memory T cells as the generation of naïve T cells in the thymus declines (91–93). Overall, immunosenescence results in an increase in susceptibility to infections, decreased vaccine effectiveness, and increased susceptibility to autoimmunity and inflammation (94). In COVID-19 patients, one study demonstrated that COVID-19 infection amplified age-associated immune changes (58). Another study found that young COVID-19 patients had greater percentages of CD8+ T cells expressing perforin, granzyme A, and granzyme B compared to healthy controls of the same age, but the same difference was not seen in elderly COVID-19 patients (41). This suggests that either cytotoxic T cell responses to SARS-CoV-2 are either inhibited in patients of advanced age or that older patients already have a higher baseline level of cytotoxic CD8+ T cells pre-infection, the latter explanation being the most consistent with previous studies. Additionally, several studies have demonstrated the increased presence of cytotoxic T cells in severe COVID-19, and recent studies suggest that the cytotoxic T cell response may be associated with the persistence of COVID-19 symptoms after the acute phase of infection (95–97). Given the important role of cytotoxic T cells in killing virus-infected cells and their potential to cause endothelial damage (97), further studies examining the functions of cytotoxic T cells in severe COVID-19 patients of all ages are prudent.

It has been suggested that increased T cell activation and inflammation are major drivers of severe COVID-19. However, our results suggest that homing ligands, specifically CD62L, may also be important indicators of severe COVID-19 requiring ICU admission. However, our study is limited in scope due to having a relatively small cohort and blood samples at only one timepoint per patient. Additionally, we have very limited information regarding the course of COVID-19 illness or medical history in our recovered and healthy cohorts, as these samples were obtained from a blood bank rather than through the hospital. Mechanistic studies are needed to confirm the tissue homing potential of CD62L+ T cells in COVID-19 and are especially necessary to determine whether pre-existing chronic conditions affect the expression of CD62L or other immune mediators. In addition, monitoring changes in CD62L expression at multiple timepoints during acute illness and recovery is critical to understand the function of CD62L during COVID-19. Such studies are critically important and may identify targets for future SARS-CoV-2 diagnostics and therapeutics.

## Methods

### Study design

Severe (admitted to ICU) and non-severe (admitted to the hospital for observation but not admitted into the ICU) COVID-19 patients were recruited as part of a prospective clinical trial of COVID-19 convalescent plasma therapy (CCP) (46). Standard ICU admission criteria for COVID included oxygen requirement >6L/min, hemodynamic instability, and/or need for higher level monitoring and nursing care in the opinion of the treating physician. Blood samples taken pre-CCP infusion were obtained from 30 randomly selected patients admitted to the University of Colorado Hospital (Aurora, CO) in April and May 2020, before the availability of COVID-19 vaccines and emergence of variants of concern. Patients admitted to this study met the following inclusion criteria: age 18 years or older, laboratory-confirmed diagnosis of SARS-CoV-2 by PCR, were admitted to the hospital for COVID-19, and informed consent was obtained. Patients were excluded if they had a history of transfusion reaction, received pooled immunoglobulin in the 30 days prior to enrollment, or if the clinician determined that the risk of CCP administration outweighed the potential benefits. All demographic and treatment data were obtained directly from the patients’ medical records. Pre-existing diagnoses of hypertension and diabetes were recorded in the patient database based on ICD-10 diagnosis codes present from the individual patient charts.

### PBMC and plasma collection

Blood from severe and non-severe COVID-19 patients was collected in 10 mL vacutainer tubes containing sodium heparin (BD) and processed for plasma and PBMCs no more than 8 hours post-collection. Blood from healthy controls (in LRS chambers) and recovered COVID-19 patients was obtained from a local blood bank (Vitalant, Denver, CO) in April-May 2020. Blood from recovered COVID-19 patients was obtained after their symptoms resolved and up to 2 months post illness. To collect plasma, the blood was centrifuged at 500 x g for 10 minutes. After removal of the plasma layer, PBMCs were isolated from the remaining blood by density gradient centrifugation using Ficoll (GE Healthcare). Viability and cell counts were determined using trypan blue (Corning). If cell viability was greater than 90%, PBMCs were cryopreserved for future experiments and thawed later for flow cytometry analysis. For cryopreservation, PBMCs were resuspended in freezing media (90% fetal bovine serum (FBS, Hyclone, Thermo Fisher) + 10% DMSO (Sigma)) at a concentration of 1-10x10^6 cells per mL, aliquoted into cryogenic vials (Corning), and placed into a Mr. Frosty container (Nalgene) at -80°C. The PBMCs were then moved into liquid nitrogen within 12 hours.

### RBD protein production and purification for ELISA

SARS-CoV-2 RBD protein was produced and purified based on previously published methods (47). Briefly, E. coli were transfected with pCAGGS plasmid containing the RBD of SARS-CoV-2 isolate Wuhan-Hu-1 (BEI Resources #NR-52309) and grown overnight in Luria-Bertani (LB) broth (GrowCells.com) containing carbenicillin (Research Products International) at 37°C. Plasmid was isolated using a Midiprep kit (Qiagen) and submitted to the University of Colorado Cancer Center Cell Technologies Shared Resource for transient transfection in Expi293 cells and purification.

### N and RBD IgG ELISAs

RBD IgG ELISAs were performed using a previously published protocol (47), except that plasma was diluted to a final concentration of 1:20. Human AB serum (Innovative Research) purchased before the COVID-19 pandemic was used as a negative control, and a commercially available rabbit anti-SARS-CoV-2 spike monoclonal antibody (catalog number 40150-R0907, Sino Biological) and donkey anti-rabbit IgG secondary antibody (Jackson ImmunoResearch) were used as a positive control. The plates were read in a VersaMax tunable microplate reader (Molecular Devices). The average of the negative control readings plus 3 times the standard deviation was used as a cut-off for positivity.

N IgG ELISAs were performed using the Elecsys Anti-SARS-CoV-2 immunoassay (Roche) on a Cobas e411 analyzer (Roche) per the manufacturer’s instructions (48). A COI value >1 was considered positive.

### Flow cytometry

Previously frozen PBMCs were thawed and rested overnight at 37°C in complete R10 media (RPMI with L-glutamine (Corning) + 10% FBS + 1% Penicillin/Streptomycin (Corning), 1% sodium pyruvate (Gibco), 1% MEM-Non-essential amino acids (Gibco), and 1% HEPES (Gibco)), and cell counts were determined using trypan blue. 0.5-1x10^6 PBMCs were aliquoted into FACS tubes (Genesee Scientific), washed with 2 mL FACS buffer (PBS (Corning) + 2% FBS), and centrifuged at 500 xg for 5 minutes. Next, Live/Dead Blue viability dye was added to the cells for 10 minutes at room temperature, followed by 50 uL of extracellular staining antibodies for 20 minutes at 4°C. The cells were then washed again in FACS buffer, and fixed and permeabilized using the Human FoxP3 Buffer Set (BD Biosciences) according to the manufacturer’s instructions. After washing in flow cytometry perm buffer (Tonbo Biosciences), the cells were incubated with 50 uL of intracellular antibodies prepared in perm buffer for 45 minutes at 4°C. After a final wash in perm buffer, the cells were fixed with 4% paraformaldehyde (PFA, Electron Microscopy Services). All antibodies used for flow cytometry are listed in **Table S2**. The samples were run on a Cytek Aurora flow cytometer. Single-color compensation controls were created by incubating antibodies with UltraComp eBeads (eBioscience) or dyes with ArC Amine Reactive Beads (Invitrogen). The data were analyzed using FlowJo software (version 10.8.0) by an investigator who was blinded to the patients’ clinical demographics. In FlowJo, cells were gated to eliminate doublets and debris, gated on lymphocytes, viable cells, CD14- CD56- CD19- cells, and CD3+ cells. CD4 and CD8 T cells were then gated, and each subset was gated on CD62L. Finally, CD62L+ T cells were subgated to measure T cell memory (CCR7 and CD45RA), Tregs (CD25+ FoxP3+ and CD25+ CD127lo), activated cells (CD25+, CD27+), CD57+ cells, and cells expressing immune checkpoint receptors (PD-1, Tim-3, CTLA-4). Fluorescence minus one (FMO) controls were used to set all flow cytometry gates, and gates were verified using multiple samples from both healthy and COVID-19 patients. FSC and SSC gates were validated by backgating on viable CD14- CD3+ cells.

### Statistical analysis

Parametric distribution testing was completed on the parent population of patients and the randomly obtained serum sample population based on white blood cell count as described previously (46). All statistical analysis was performed in GraphPad Prism version 9.2.0. One-Way ANOVA with Tukey’s multiple comparisons test was used to compare results between 4 groups, and a t test was used to compare results between 2 groups. Categorical data were compared using Fisher’s exact test. A p value <0.05 was considered statistically significant. When outliers were present, the ROUT method (Q value of 1%) was used to remove them. The data are represented as mean ± standard deviation, and each symbol on a graph represents one patient.

## Data availability statement

The raw data supporting the conclusions of this article will be made available by the authors, without undue reservation.

## Ethics statement

The studies involving human participants were reviewed and approved by Colorado Multiple Institutional Review Board. Written informed consent to participate was obtained either directly from the patient, patient proxy, or durable power of attorney.

## Author contributions

KL designed the flow cytometry studies, collected and analyzed data, and wrote the manuscript; PA and KW assisted with flow cytometry panel design, analyzed data, and reviewed the manuscript; LC conducted the clinical trial, provided blood samples, and reviewed the manuscript; JE provided funding and reviewed the manuscript; and JB provided funding, designed clinical studies, acquired clinical data, and wrote the manuscript. All authors contributed to the article and approved the submitted version

## References

[1] Wu F Zhao S Yu B Chen YM Wang W Song ZG. A new coronavirus associated with human respiratory disease in China. Nature (2020) 579(7798):265–9. doi: 10.1038/s41586-020-2008-3 PMC7094943 32015508

[2] Zhou P Yang XL Wang XG Hu B Zhang L Zhang W. A pneumonia outbreak associated with a new coronavirus of probable bat origin. Nature (2020) 579(7798):270–3. doi: 10.1038/s41586-020-2012-7 PMC7095418 32015507

[3] Huang C Wang Y Li X Ren L Zhao J Hu Y. Clinical features of patients infected with 2019 novel coronavirus in wuhan, China. Lancet (2020) 395(10223):497–506. doi: 10.1016/S0140-6736(20)30183-5 31986264 PMC7159299

[4] Qin C Zhou L Hu Z Zhang S Yang S Tao Y. Dysregulation of immune response in patients with coronavirus 2019 (COVID-19) in wuhan, China. Clin Infect Dis (2020) 71(15):762–8. doi: 10.1093/cid/ciaa248 PMC7108125 32161940

[5] Guan WJ Ni ZY Hu Y Liang WH Ou CQ He JX. Clinical characteristics of coronavirus disease 2019 in China. N Engl J Med (2020) 382(18):1708–20. doi: 10.1056/NEJMoa2002032 PMC7092819 32109013

[6] Wu Z McGoogan JM. Characteristics of and important lessons from the coronavirus disease 2019 (COVID-19) outbreak in China: Summary of a report of 72314 cases from the Chinese center for disease control and prevention. JAMA (2020) 323(13):1239–42. doi: 10.1001/jama.2020.2648 32091533

[7] Docherty AB Harrison EM Green CA Hardwick HE Pius R Norman L. Features of 20 133 UK patients in hospital with covid-19 using the ISARIC WHO clinical characterisation protocol: prospective observational cohort study. BMJ (2020) 369:m1985. doi: 10.1136/bmj.m1985 32444460 PMC7243036

[8] Liu K Chen Y Lin R Han K. Clinical features of COVID-19 in elderly patients: A comparison with young and middle-aged patients. J Infect (2020) 80(6):e14–e8. doi: 10.1016/j.jinf.2020.03.005 PMC7102640 32171866

[9] Kompaniyets L Goodman AB Belay B Freedman DS Sucosky MS Lange SJ. Body mass index and risk for COVID-19-Related hospitalization, intensive care unit admission, invasive mechanical ventilation, and death - united states, march-December 2020. MMWR Morb Mortal Wkly Rep (2021) 70(10):355–61. doi: 10.15585/mmwr.mm7010e4 PMC7951819 33705371

[10] Liu C Zhao Y Okwan-Duodu D Basho R Cui X. COVID-19 in cancer patients: risk, clinical features, and management. Cancer Biol Med (2020) 17(3):519–27. doi: 10.20892/j.issn.2095-3941.2020.0289 PMC7476081 32944387

[11] Zhang L Zhu F Xie L Wang C Wang J Chen R. Clinical characteristics of COVID-19-infected cancer patients: a retrospective case study in three hospitals within wuhan, China. Ann Oncol (2020) 31(7):894–901. doi: 10.1016/j.annonc.2020.03.296 32224151 PMC7270947

[12] Andersen KM Bates BA Rashidi ES Olex AL Mannon RB Patel RC. Long-term use of immunosuppressive medicines and in-hospital COVID-19 outcomes: a retrospective cohort study using data from the national COVID cohort collaborative. Lancet Rheumatol (2022) 4(1):e33–41. doi: 10.1016/S2665-9913(21)00325-8 PMC8592562 34806036

[13] Garcia LF. Immune response, inflammation, and the clinical spectrum of COVID-19. Front Immunol (2020) 11:1441. doi: 10.3389/fimmu.2020.01441 32612615 PMC7308593

[14] Braun J Loyal L Frentsch M Wendisch D Georg P Kurth F. SARS-CoV-2-reactive T cells in healthy donors and patients with COVID-19. Nature (2020) 587(7833):270–4. doi: 10.1038/s41586-020-2598-9 32726801

[15] Grifoni A Weiskopf D Ramirez SI Mateus J Dan JM Moderbacher CR. Targets of T cell responses to SARS-CoV-2 coronavirus in humans with COVID-19 disease and unexposed individuals. Cell (2020) 181(7):1489–501 e15. doi: 10.1016/j.cell.2020.05.015 32473127 PMC7237901

[16] Kared H Redd AD Bloch EM Bonny TS Sumatoh H Kairi F. SARS-CoV-2-specific CD8+ T cell responses in convalescent COVID-19 individuals. J Clin Invest (2021) 131(5):e145476. doi: 10.1172/JCI145476 33427749 PMC7919723

[17] Zhao J Zhao J Mangalam AK Channappanavar R Fett C Meyerholz DK. Airway memory CD4(+) T cells mediate protective immunity against emerging respiratory coronaviruses. Immunity (2016) 44(6):1379–91. doi: 10.1016/j.immuni.2016.05.006 PMC4917442 27287409

[18] Zhao J Zhao J Perlman S. T Cell responses are required for protection from clinical disease and for virus clearance in severe acute respiratory syndrome coronavirus-infected mice. J Virol (2010) 84(18):9318–25. doi: 10.1128/JVI.01049-10 PMC2937604 20610717

[19] Schmidt ME Varga SM. The CD8 T cell response to respiratory virus infections. Front Immunol (2018) 9:678. doi: 10.3389/fimmu.2018.00678 29686673 PMC5900024

[20] Kervevan J Chakrabarti LA. Role of CD4+ T cells in the control of viral infections: Recent advances and open questions. Int J Mol Sci (2021) 22(2):523. doi: 10.3390/ijms22020523 33430234 PMC7825705

[21] Swain SL McKinstry KK Strutt TM. Expanding roles for CD4(+) T cells in immunity to viruses. Nat Rev Immunol (2012) 12(2):136–48. doi: 10.1038/nri3152 PMC3764486 22266691

[22] Voskoboinik I Smyth MJ Trapani JA. Perforin-mediated target-cell death and immune homeostasis. Nat Rev Immunol (2006) 6(12):940–52. doi: 10.1038/nri1983 17124515

[23] Halle S Halle O Forster R. Mechanisms and dynamics of T cell-mediated cytotoxicity in vivo. Trends Immunol (2017) 38(6):432–43. doi: 10.1016/j.it.2017.04.002 28499492

[24] Toor SM Saleh R Sasidharan Nair V Taha RZ Elkord E. T-Cell responses and therapies against SARS-CoV-2 infection. Immunology (2021) 162(1):30–43. doi: 10.1111/imm.13262 32935333 PMC7730020

[25] Zhou Y Fu B Zheng X Wang D Zhao C Qi Y. Pathogenic T-cells and inflammatory monocytes incite inflammatory storms in severe COVID-19 patients. Natl Sci Rev (2020) 7(6):998–1002. doi: 10.1093/nsr/nwaa041 34676125 PMC7108005

[26] Le Bert N Tan AT Kunasegaran K Tham CYL Hafezi M Chia A. SARS-CoV-2-specific T cell immunity in cases of COVID-19 and SARS, and uninfected controls. Nature (2020) 584(7821):457–62. doi: 10.1038/s41586-020-2550-z 32668444

[27] Sattler A Angermair S Stockmann H Heim KM Khadzhynov D Treskatsch S. SARS-CoV-2-specific T cell responses and correlations with COVID-19 patient predisposition. J Clin Invest (2020) 130(12):6477–89. doi: 10.1172/JCI140965 PMC7685725 32833687

[28] Garcia-Gonzalez P Tempio F Fuentes C Merino C Vargas L Simon V. Dysregulated immune responses in COVID-19 patients correlating with disease severity and invasive oxygen requirements. Front Immunol (2021) 12:769059. doi: 10.3389/fimmu.2021.769059 34745145 PMC8567168

[29] Meckiff BJ Ramirez-Suastegui C Fajardo V Chee SJ Kusnadi A Simon H. Imbalance of regulatory and cytotoxic SARS-CoV-2-Reactive CD4(+) T cells in COVID-19. Cell (2020) 183(5):1340–53 e16. doi: 10.1016/j.cell.2020.10.001 33096020 PMC7534589

[30] Shah VK Firmal P Alam A Ganguly D Chattopadhyay S. Overview of immune response during SARS-CoV-2 infection: Lessons from the past. Front Immunol (2020) 11:1949. doi: 10.3389/fimmu.2020.01949 32849654 PMC7426442

[31] Anderson AC Joller N Kuchroo VK. Lag-3, Tim-3, and TIGIT: Co-inhibitory receptors with specialized functions in immune regulation. Immunity (2016) 44(5):989–1004. doi: 10.1016/j.immuni.2016.05.001 27192565 PMC4942846

[32] Tang R Rangachari M Kuchroo VK. Tim-3: A co-receptor with diverse roles in T cell exhaustion and tolerance. Semin Immunol (2019) 42:101302. doi: 10.1016/j.smim.2019.101302 31604535

[33] Buchbinder EI Desai A. CTLA-4 and PD-1 pathways: Similarities, differences, and implications of their inhibition. Am J Clin Oncol (2016) 39(1):98–106. doi: 10.1097/COC.0000000000000239 26558876 PMC4892769

[34] Sun C Mezzadra R Schumacher TN. Regulation and function of the PD-L1 checkpoint. Immunity (2018) 48(3):434–52. doi: 10.1016/j.immuni.2018.03.014 PMC7116507 29562194

[35] D'Alessio FR Tsushima K Aggarwal NR West EE Willett MH Britos MF. CD4+CD25+Foxp3+ tregs resolve experimental lung injury in mice and are present in humans with acute lung injury. J Clin Invest (2009) 119(10):2898–913. doi: 10.1172/JCI36498 PMC2752062 19770521

[36] Mangodt TC Van Herck MA Nullens S Ramet J De Dooy JJ Jorens PG. The role of Th17 and treg responses in the pathogenesis of RSV infection. Pediatr Res (2015) 78(5):483–91. doi: 10.1038/pr.2015.143 26267154

[37] Antunes I Kassiotis G. Suppression of innate immune pathology by regulatory T cells during influenza a virus infection of immunodeficient mice. J Virol (2010) 84(24):12564–75. doi: 10.1128/JVI.01559-10 PMC3004299 20943986

[38] Rha MS Jeong HW Ko JH Choi SJ Seo IH Lee JS. PD-1-Expressing SARS-CoV-2-Specific CD8(+) T cells are not exhausted, but functional in patients with COVID-19. Immunity (2021) 54(1):44–52 e3. doi: 10.1016/j.immuni.2020.12.002 33338412 PMC7834198

[39] Diao B Wang C Tan Y Chen X Liu Y Ning L. Reduction and functional exhaustion of T cells in patients with coronavirus disease 2019 (COVID-19). Front Immunol (2020) 11:827. doi: 10.3389/fimmu.2020.00827 32425950 PMC7205903

[40] Kusnadi A Ramirez-Suastegui C Fajardo V Chee SJ Meckiff BJ Simon H. Severely ill COVID-19 patients display impaired exhaustion features in SARS-CoV-2-reactive CD8(+) T cells. Sci Immunol (2021) 6(55):eabe4782. doi: 10.1126/sciimmunol.abe4782 33478949 PMC8101257

[41] Westmeier J Paniskaki K Karakose Z Werner T Sutter K Dolff S. Impaired cytotoxic CD8(+) T cell response in elderly COVID-19 patients. mBio (2020) 11(5):e02243-20. doi: 10.1128/mBio.02243-20 32948688 PMC7502863

[42] Zheng HY Zhang M Yang CX Zhang N Wang XC Yang XP. Elevated exhaustion levels and reduced functional diversity of T cells in peripheral blood may predict severe progression in COVID-19 patients. Cell Mol Immunol (2020) 17(5):541–3. doi: 10.1038/s41423-020-0401-3 PMC7091621 32203186

[43] Arbones ML Ord DC Ley K Ratech H Maynard-Curry C Otten G. Lymphocyte homing and leukocyte rolling and migration are impaired in l-selectin-deficient mice. Immunity (1994) 1(4):247–60. doi: 10.1016/1074-7613(94)90076-0 7534203

[44] Galkina E Kadl A Sanders J Varughese D Sarembock IJ Ley K. Lymphocyte recruitment into the aortic wall before and during development of atherosclerosis is partially l-selectin dependent. J Exp Med (2006) 203(5):1273–82. doi: 10.1084/jem.20052205 PMC2121208 16682495

[45] Mohammed RN Watson HA Vigar M Ohme J Thomson A Humphreys IR. L-selectin is essential for delivery of activated CD8(+) T cells to virus-infected organs for protective immunity. Cell Rep (2016) 14(4):760–71. doi: 10.1016/j.celrep.2015.12.090 PMC4742564 26804910

[46] Chauhan L Pattee J Ford J Thomas C Lesteberg K Richards E. A multi-center, prospective, observational-cohort controlled study of clinical outcomes following COVID-19 convalescent plasma therapy in hospitalized COVID-19 patients. Clin Infect Dis (2021) 75(1):e466–e472. doi: 10.1101/2021.06.14.21258910 PMC9612788 34549274

[47] Stadlbauer D Amanat F Chromikova V Jiang K Strohmeier S Arunkumar GA. SARS-CoV-2 seroconversion in humans: A detailed protocol for a serological assay, antigen production, and test setup. Curr Protoc Microbiol (2020) 57(1):e100. doi: 10.1002/cpmc.100 32302069 PMC7235504

[48] Riester E Majchrzak M Muhlbacher A Tinguely C Findeisen P Hegel JK. Multicentre performance evaluation of the elecsys anti-SARS-CoV-2 immunoassay as an aid in determining previous exposure to SARS-CoV-2. Infect Dis Ther (2021) 10(4):2381–97. doi: 10.1007/s40121-021-00504-9 PMC8349665 34368915

[49] Baang JH Smith C Mirabelli C Valesano AL Manthei DM Bachman MA. Prolonged severe acute respiratory syndrome coronavirus 2 replication in an immunocompromised patient. J Infect Dis (2021) 223(1):23–7. doi: 10.1093/infdis/jiaa666 PMC7797758 33089317

[50] Tang L Cherry S Tuomanen EI Roubidoux EK Lin CY Allison KJ. Host predictors of broadly cross-reactive antibodies against SARS-CoV-2 variants of concern differ between infection and vaccination. Clin Infect Dis (2021) 75(1):e705–e714. doi: 10.1093/cid/ciab996 PMC8689782 34891165

[51] Ivetic A Hoskins Green HL Hart SJ. L-selectin: A major regulator of leukocyte adhesion, migration and signaling. Front Immunol (2019) 10:1068. doi: 10.3389/fimmu.2019.01068 31139190 PMC6527602

[52] Karlsson AC Humbert M Buggert M. The known unknowns of T cell immunity to COVID-19. Sci Immunol (2020) 5(53):eabe8063. doi: 10.1126/sciimmunol.abe8063 33208380

[53] Thevarajan I Nguyen THO Koutsakos M Druce J Caly L van de Sandt CE. Breadth of concomitant immune responses prior to patient recovery: a case report of non-severe COVID-19. Nat Med (2020) 26(4):453–5. doi: 10.1038/s41591-020-0819-2 PMC7095036 32284614

[54] Sekine T Perez-Potti A Rivera-Ballesteros O Stralin K Gorin JB Olsson A. Robust T cell immunity in convalescent individuals with asymptomatic or mild COVID-19. Cell (2020) 183(1):158–68 e14. doi: 10.1016/j.cell.2020.08.017 32979941 PMC7427556

[55] Kared H Martelli S Ng TP Pender SL Larbi A. CD57 in human natural killer cells and T-lymphocytes. Cancer Immunol Immunother (2016) 65(4):441–52. doi: 10.1007/s00262-016-1803-z PMC11029668 26850637

[56] Lefrancois L Marzo AL. The descent of memory T-cell subsets. Nat Rev Immunol (2006) 6(8):618–23. doi: 10.1038/nri1866 16868553

[57] Yao WL Wen Q Zhao HY Tang SQ Zhang YY Wang Y. Different subsets of haematopoietic cells and immune cells in bone marrow between young and older donors. Clin Exp Immunol (2021) 203(1):137–49. doi: 10.1111/cei.13531 PMC7744501 33020903

[58] Zheng Y Liu X Le W Xie L Li H Wen W. A human circulating immune cell landscape in aging and COVID-19. Protein Cell (2020) 11(10):740–70. doi: 10.1007/s13238-020-00762-2 PMC7417788 32780218

[59] Gao YD Ding M Dong X Zhang JJ Kursat Azkur A Azkur D. Risk factors for severe and critically ill COVID-19 patients: A review. Allergy (2021) 76(2):428–55. doi: 10.1111/all.14657 33185910

[60] Myers LC Parodi SM Escobar GJ Liu VX. Characteristics of hospitalized adults with COVID-19 in an integrated health care system in California. JAMA (2020) 323(21):2195–8. doi: 10.1001/jama.2020.7202 PMC7182961 32329797

[61] Rosenberg C Bovin NV Bram LV Flyvbjerg E Erlandsen M Vorup-Jensen T. Age is an important determinant in humoral and T cell responses to immunization with hepatitis b surface antigen. Hum Vaccin Immunother (2013) 9(7):1466–76. doi: 10.4161/hv.24480 23571167

[62] Peng YD Meng K Guan HQ Leng L Zhu RR Wang BY. [Clinical characteristics and outcomes of 112 cardiovascular disease patients infected by 2019-nCoV]. Zhonghua Xin Xue Guan Bing Za Zhi (2020) 48(6):450–5. doi: 10.3760/cma.j.cn112148-20200220-00105 32120458

[63] Petrilli CM Jones SA Yang J Rajagopalan H O'Donnell L Chernyak Y. Factors associated with hospital admission and critical illness among 5279 people with coronavirus disease 2019 in new York city: prospective cohort study. BMJ (2020) 369:m1966. doi: 10.1136/bmj.m1966 32444366 PMC7243801

[64] Simonnet A Chetboun M Poissy J Raverdy V Noulette J Duhamel A. High prevalence of obesity in severe acute respiratory syndrome coronavirus-2 (SARS-CoV-2) requiring invasive mechanical ventilation. Obes (Silver Spring) (2020) 28(7):1195–9. doi: 10.1002/oby.22831 PMC7262326 32271993

[65] Misumi I Starmer J Uchimura T Beck MA Magnuson T Whitmire JK. Obesity expands a distinct population of T cells in adipose tissue and increases vulnerability to infection. Cell Rep (2019) 27(2):514–24 e5. doi: 10.1016/j.celrep.2019.03.030 30970254 PMC6652206

[66] SantaCruz-Calvo S Bharath L Pugh G SantaCruz-Calvo L Lenin RR Lutshumba J. Adaptive immune cells shape obesity-associated type 2 diabetes mellitus and less prominent comorbidities. Nat Rev Endocrinol (2022) 18(1):23–42. doi: 10.1038/s41574-021-00575-1 34703027 PMC11005058

[67] Mills KT Stefanescu A He J. The global epidemiology of hypertension. Nat Rev Nephrol (2020) 16(4):223–37. doi: 10.1038/s41581-019-0244-2 PMC7998524 32024986

[68] Khan MAB Hashim MJ King JK Govender RD Mustafa H Al Kaabi J. Epidemiology of type 2 diabetes - global burden of disease and forecasted trends. J Epidemiol Glob Health (2020) 10(1):107–11. doi: 10.2991/jegh.k.191028.001 PMC7310804 32175717

[69] Lim S Bae JH Kwon HS Nauck MA. COVID-19 and diabetes mellitus: from pathophysiology to clinical management. Nat Rev Endocrinol (2021) 17(1):11–30. doi: 10.1038/s41574-020-00435-4 33188364 PMC7664589

[70] Gray TJ Webb CE. A review of the epidemiological and clinical aspects of West Nile virus. Int J Gen Med (2014) 7:193–203. doi: 10.2147/IJGM.S59902 24748813 PMC3990373

[71] Montgomery RR Murray KO. Risk factors for West Nile virus infection and disease in populations and individuals. Expert Rev Anti Infect Ther (2015) 13(3):317–25. doi: 10.1586/14787210.2015.1007043 PMC4939899 25637260

[72] Singh AK Gupta R Ghosh A Misra A. Diabetes in COVID-19: Prevalence, pathophysiology, prognosis and practical considerations. Diabetes Metab Syndr (2020) 14(4):303–10. doi: 10.1016/j.dsx.2020.04.004 PMC7195120 32298981

[73] Muller LM Gorter KJ Hak E Goudzwaard WL Schellevis FG Hoepelman AI. Increased risk of common infections in patients with type 1 and type 2 diabetes mellitus. Clin Infect Dis (2005) 41(3):281–8. doi: 10.1086/431587 16007521

[74] Yang XD Karin N Tisch R Steinman L McDevitt HO. Inhibition of insulitis and prevention of diabetes in nonobese diabetic mice by blocking l-selectin and very late antigen 4 adhesion receptors. Proc Natl Acad Sci U S A (1993) 90(22):10494–8. doi: 10.1073/pnas.90.22.10494 PMC47803 7504266

[75] Lepault F Gagnerault MC Faveeuw C Bazin H Boitard C. Lack of l-selectin expression by cells transferring diabetes in NOD mice: insights into the mechanisms involved in diabetes prevention by Mel-14 antibody treatment. Eur J Immunol (1995) 25(6):1502–7. doi: 10.1002/eji.1830250605 7542194

[76] Friedline RH Wong CP Steeber DA Tedder TF Tisch R. L-selectin is not required for T cell-mediated autoimmune diabetes. J Immunol (2002) 168(6):2659–66. doi: 10.4049/jimmunol.168.6.2659 11884430

[77] Martin MD Badovinac VP. Defining memory CD8 T cell. Front Immunol (2018) 9:2692. doi: 10.3389/fimmu.2018.02692 30515169 PMC6255921

[78] Sallusto F Lenig D Forster R Lipp M Lanzavecchia A. Two subsets of memory T lymphocytes with distinct homing potentials and effector functions. Nature (1999) 401(6754):708–12. doi: 10.1038/44385 10537110

[79] Mora JR von Andrian UH. T-Cell homing specificity and plasticity: new concepts and future challenges. Trends Immunol (2006) 27(5):235–43. doi: 10.1016/j.it.2006.03.007 16580261

[80] Brownlie D Rodahl I Varnaite R Asgeirsson H Glans H Falck-Jones S. Comparison of lung-homing receptor expression and activation profiles on NK cell and T cell subsets in COVID-19 and influenza. Front Immunol (2022) 13:834862. doi: 10.3389/fimmu.2022.834862 35371005 PMC8966396

[81] Chua RL Lukassen S Trump S Hennig BP Wendisch D Pott F. COVID-19 severity correlates with airway epithelium-immune cell interactions identified by single-cell analysis. Nat Biotechnol (2020) 38(8):970–9. doi: 10.1038/s41587-020-0602-4 32591762

[82] Wolf Y Anderson AC Kuchroo VK. TIM3 comes of age as an inhibitory receptor. Nat Rev Immunol (2020) 20(3):173–85. doi: 10.1038/s41577-019-0224-6 PMC7327798 31676858

[83] Kasprowicz V Schulze Zur Wiesch J Kuntzen T Nolan BE Longworth S Berical A. High level of PD-1 expression on hepatitis c virus (HCV)-specific CD8+ and CD4+ T cells during acute HCV infection, irrespective of clinical outcome. J Virol (2008) 82(6):3154–60. doi: 10.1128/JVI.02474-07 PMC2258997 18160439

[84] Zhang Z Zhang JY Wherry EJ Jin B Xu B Zou ZS. Dynamic programmed death 1 expression by virus-specific CD8 T cells correlates with the outcome of acute hepatitis b. Gastroenterology (2008) 134(7):1938–49, 49 e1-3. doi: 10.1053/j.gastro.2008.03.037 18455515

[85] Viurcos-Sanabria R Manjarrez-Reyna AN Solleiro-Villavicencio H Rizo-Tellez SA Mendez-Garcia LA Viurcos-Sanabria V. *In vitro* exposure of primary human T cells and monocytes to polyclonal stimuli reveals a basal susceptibility to display an impaired cellular immune response and develop severe COVID-19. Front Immunol (2022) 13:897995. doi: 10.3389/fimmu.2022.897995 35860236 PMC9289744

[86] Fu S Yopp AC Mao X Chen D Zhang N Chen D. CD4+ CD25+ CD62+ T-regulatory cell subset has optimal suppressive and proliferative potential. Am J Transpl (2004) 4(1):65–78. doi: 10.1046/j.1600-6143.2003.00293.x 14678036

[87] Ermann J Hoffmann P Edinger M Dutt S Blankenberg FG Higgins JP. Only the CD62L+ subpopulation of CD4+CD25+ regulatory T cells protects from lethal acute GVHD. Blood (2005) 105(5):2220–6. doi: 10.1182/blood-2004-05-2044 15546950

[88] Yang J Zhong M Hong K Yang Q Zhang E Zhou D. Characteristics of T-cell responses in COVID-19 patients with prolonged SARS-CoV-2 positivity - a cohort study. Clin Transl Immunol (2021) 10(3):e1259. doi: 10.1002/cti2.1259 PMC7932004 33728049

[89] Thompson WW Shay DK Weintraub E Brammer L Cox N Anderson LJ. Mortality associated with influenza and respiratory syncytial virus in the united states. JAMA (2003) 289(2):179–86. doi: 10.1001/jama.289.2.179 12517228

[90] Montgomery RR. Age-related alterations in immune responses to West Nile virus infection. Clin Exp Immunol (2017) 187(1):26–34. doi: 10.1111/cei.12863 27612657 PMC5167051

[91] Haynes BF Markert ML Sempowski GD Patel DD Hale LP. The role of the thymus in immune reconstitution in aging, bone marrow transplantation, and HIV-1 infection. Annu Rev Immunol (2000) 18:529–60. doi: 10.1146/annurev.immunol.18.1.529 10837068

[92] Hakim FT Gress RE. Immunosenescence: deficits in adaptive immunity in the elderly. Tissue Antigens (2007) 70(3):179–89. doi: 10.1111/j.1399-0039.2007.00891.x 17661905

[93] Zhang H Weyand CM Goronzy JJ Gustafson CE. Understanding T cell aging to improve anti-viral immunity. Curr Opin Virol (2021) 51:127–33. doi: 10.1016/j.coviro.2021.09.017 PMC8643327 34688983

[94] Pawelec G. Age and immunity: What is "immunosenescence"? Exp Gerontol (2018) 105:4–9. doi: 10.1016/j.exger.2017.10.024 29111233

[95] Kundura L Cezar R Andre S Campos-Mora M Lozano C Vincent T. Low perforin expression in CD8+ T lymphocytes during the acute phase of severe SARS-CoV-2 infection predicts long COVID. Front Immunol (2022) 13:1029006. doi: 10.3389/fimmu.2022.1029006 36341327 PMC9630742

[96] Vijayakumar B Boustani K Ogger PP Papadaki A Tonkin J Orton CM. Immuno-proteomic profiling reveals aberrant immune cell regulation in the airways of individuals with ongoing post-COVID-19 respiratory disease. Immunity (2022) 55(3):542–56 e5. doi: 10.1016/j.immuni.2022.01.017 35151371 PMC8789571

[97] Georg P Astaburuaga-Garcia R Bonaguro L Brumhard S Michalick L Lippert LJ. Complement activation induces excessive T cell cytotoxicity in severe COVID-19. Cell (2022) 185(3):493–512 e25. doi: 10.1016/j.cell.2021.12.040 35032429 PMC8712270

