# Supplementary material for: Severely ill and high-risk COVID-19 patients exhibit increased peripheral circulation of CD62L+ and perforin+ T cells
Source: Front Immunol. 2023 Feb 2;14:1113932. doi: 10.3389/fimmu.2023.1113932 (PMC9932815; doi:10.3389/fimmu.2023.1113932)
Supplement: Supplementary file 1 [file DataSheet_1.docx]

***Supplementary Material***

| **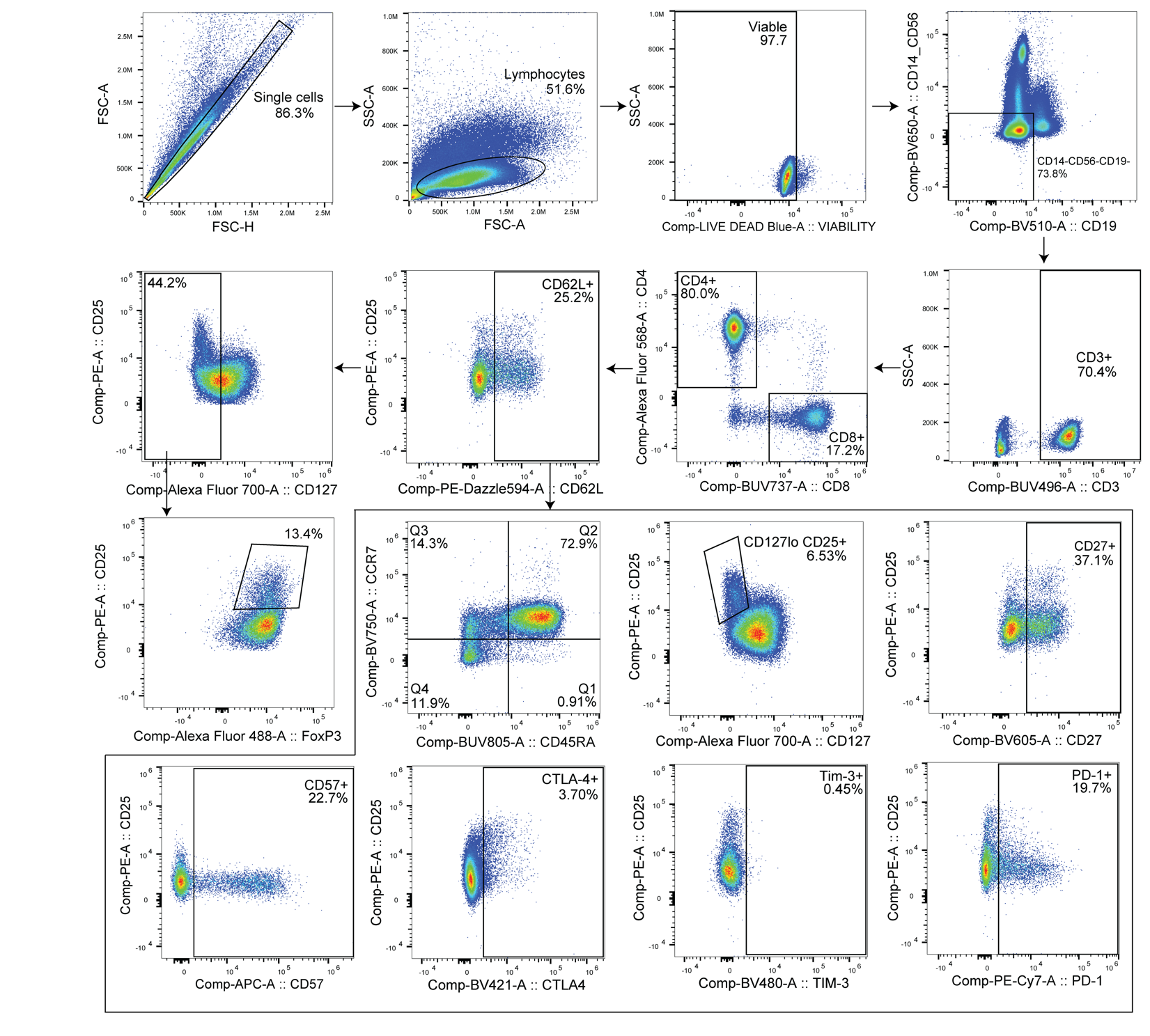** |
| --- |

**Figure S1. Flow cytometry gating strategy**. Cells were gated to eliminate doublets and debris, gated on lymphocytes, viable cells, CD14- CD56- CD19- cells, and CD3+ cells. CD4 and CD8 T cells were then gated, and each subset was gated on CD62L. To measure CD62L+ Tregs, CD62L+ T cells were gated on CD127lo and then on CD25+ FoxP3+. Finally, CD62L+ T cells were subgated to measure T cell memory (CCR7 and CD45RA), Tregs (CD25+ FoxP3+ and CD25+ CD127lo), activated cells (CD25+, CD27+), CD57+ cells, and cells expressing immune checkpoint receptors (PD-1, Tim-3, CTLA-4). Gates were determined using fluorescence minus one (FMO) controls, and the same gates were applied to all samples.

| 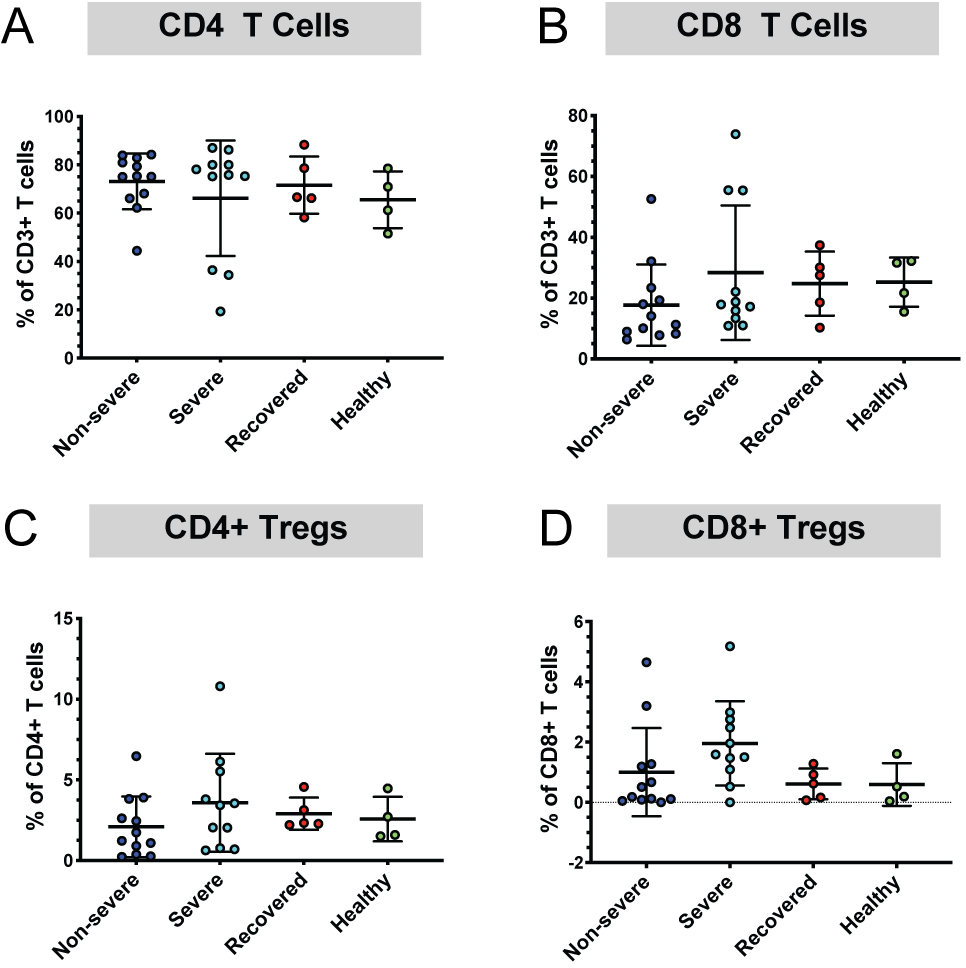 |
| --- |

**Figure S2. Frequencies of total CD4 and CD8 T cells and Tregs in COVID-19 patients.** The total percentages of CD4+ and CD8+ T cells and Tregs (CD4+ CD25+ FoxP3+ in non-severe and severe COVID-19 patients, recovered COVID-19 patients, and healthy controls were assessed by spectral flow cytometry. Cells were gated to eliminate doublets and debris, gated on lymphocytes, viable cells, CD14- CD56- CD19- cells, and CD3+ cells. A) Frequencies of CD4+ T cells, B) frequencies of CD8+ T cells, C) frequencies of CD4+ Tregs, D) frequencies of CD8+ Tregs (expressed as percentages of total CD3+ (A-B), CD4+ (C), or CD8+ cells (D)).

| 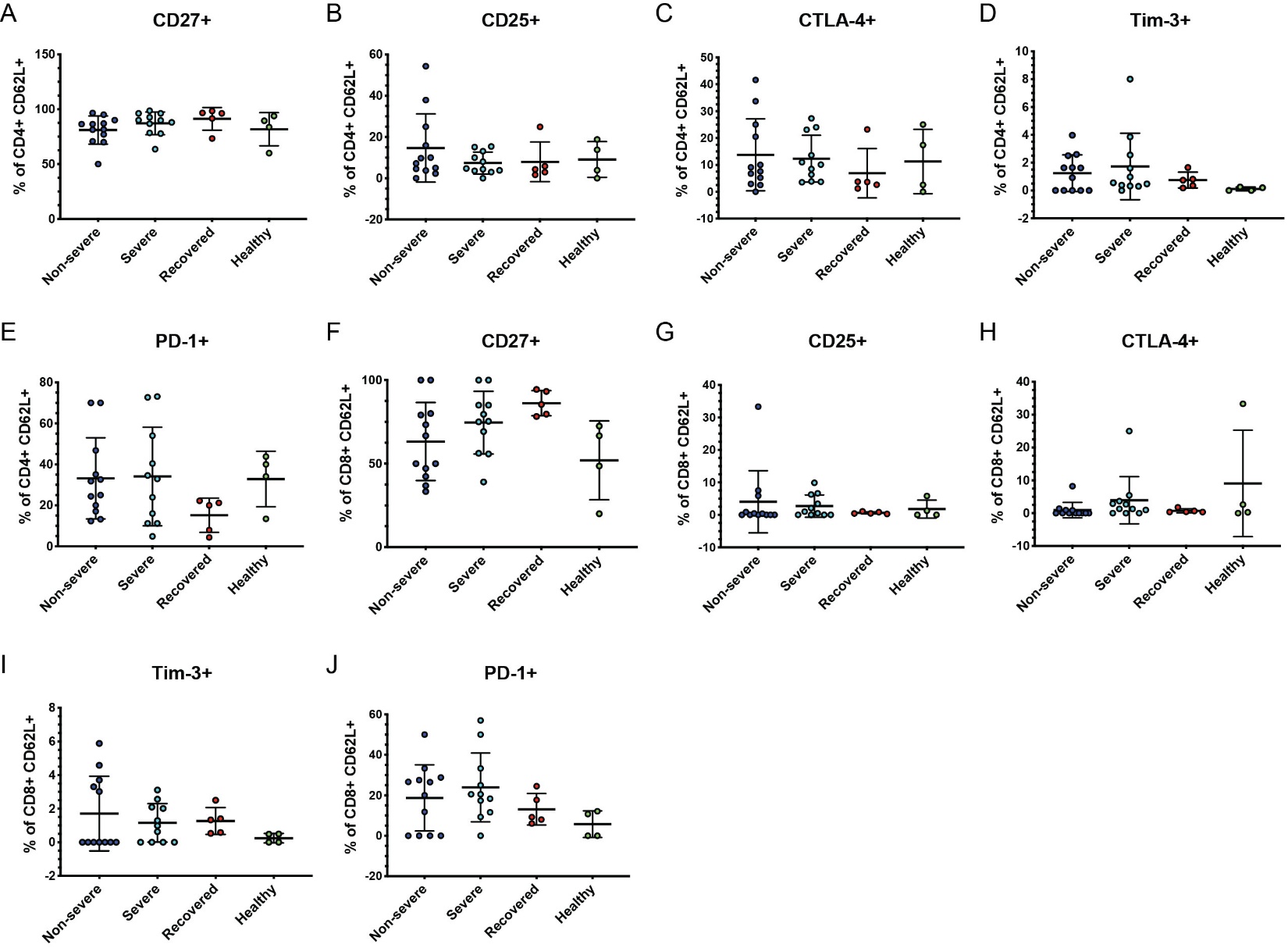 |
| --- |

**Figure S3. Expression of activation and inhibitory markers on CD62L+ T cells.** The frequencies of cells expressing CD27 (A), CD25 (B), CTLA-4 (C), Tim-3 (D), and PD-1 (E) within the CD4+ CD62L+ population and the frequencies of cells expressing CD27 (F), CD25 (G), CTLA-4 (H), Tim-3 (I), and PD-1 (J) within the CD8+ CD62L+ population in non-severe and severe COVID-19 patients, recovered COVID-19 patients, and healthy controls were assessed by spectral flow cytometry. Cells were gated on viable lymphocytes, CD14-, CD56-, CD19-, CD3+, CD4+ or CD8+ cells, CD62L+, and activation or inhibitory markers. Data are graphed as percentages of the total CD4+ CD62L+ or CD8+ CD62L+ T cell population.

| 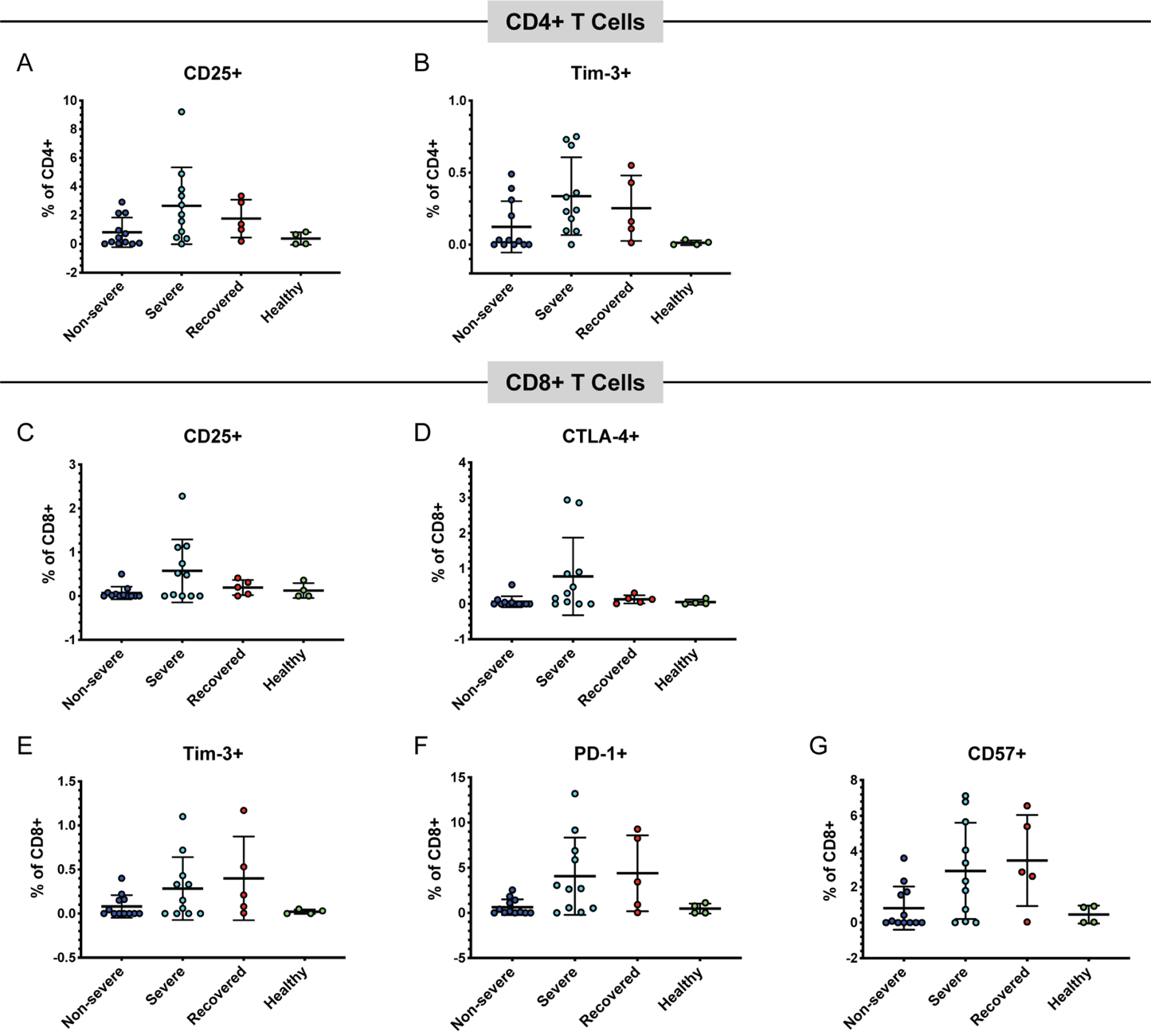 |
| --- |

**Figure S4. Frequencies of CD62L+ T cells expressing activation or inhibitory markers.** The frequencies of CD4+ CD62L+ CD25+ (A) and CD4+ CD62L+ Tim-3+ (B) T cells within the CD4+ population; CD8+ CD62L+ CD25+ (C), CD8+ CD62L+ CTLA-4+ (D), CD8+ CD62L+ Tim-3+ (E), CD8+ CD62L+ PD-1+ (F), and CD8+ CD62L+ CD57+ T cells within the CD8+ population in non-severe and severe COVID-19 patients, recovered COVID-19 patients, and healthy controls were assessed by spectral flow cytometry. Cells were gated on viable lymphocytes, CD14-, CD56-, CD19-, CD3+, CD4+ or CD8+ cells, CD62L+, and activation or inhibitory markers.

| 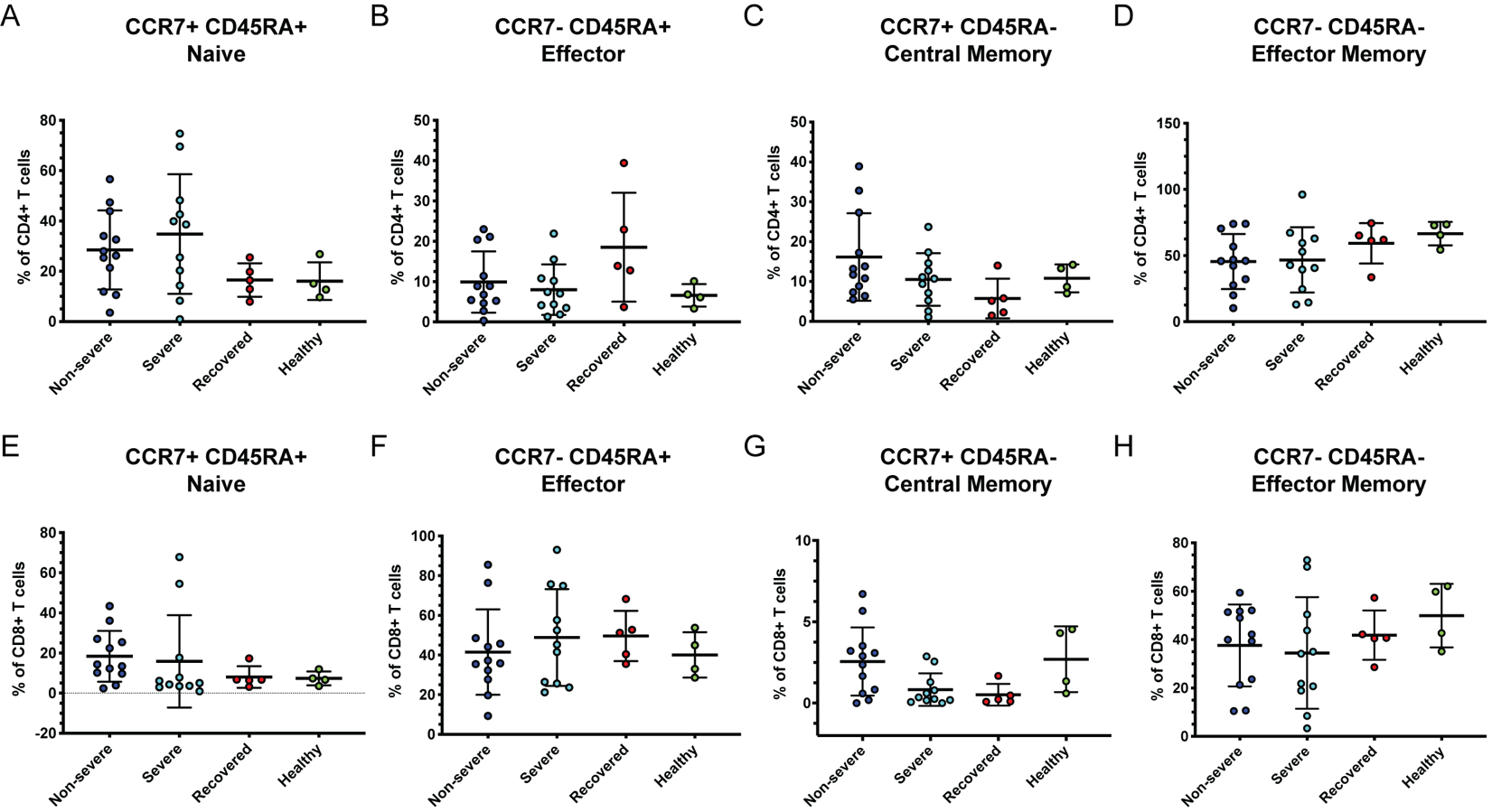 |
| --- |

**Figure S5. Analysis of T cell subsets in COVID-19 patients.** Expression of CCR7 and CD45RA on total CD4 and CD8 T cells in non-severe and severe COVID-19 patients, recovered COVID-19 patients, and healthy controls was assessed by spectral flow cytometry. (A) Percentage of naïve (CCR7+ CD45RA+), (B) effector/Temra (CCR7- CD45RA+), (C) central memory (CCR7+ CD45RA-), and (D) effector memory (CCR7- CD45RA-) cells within the CD4+ T cell population; (E) Percentage of naïve, (F) effector/Temra, (G) central memory, and (H) effector memory cells within the CD8+ T cells population. Cells were gated on viable lymphocytes, CD14-, CD56-, CD19-, CD3+, CD4+ or CD8+ cells, CD62L+, and CCR7/CD45RA.

| 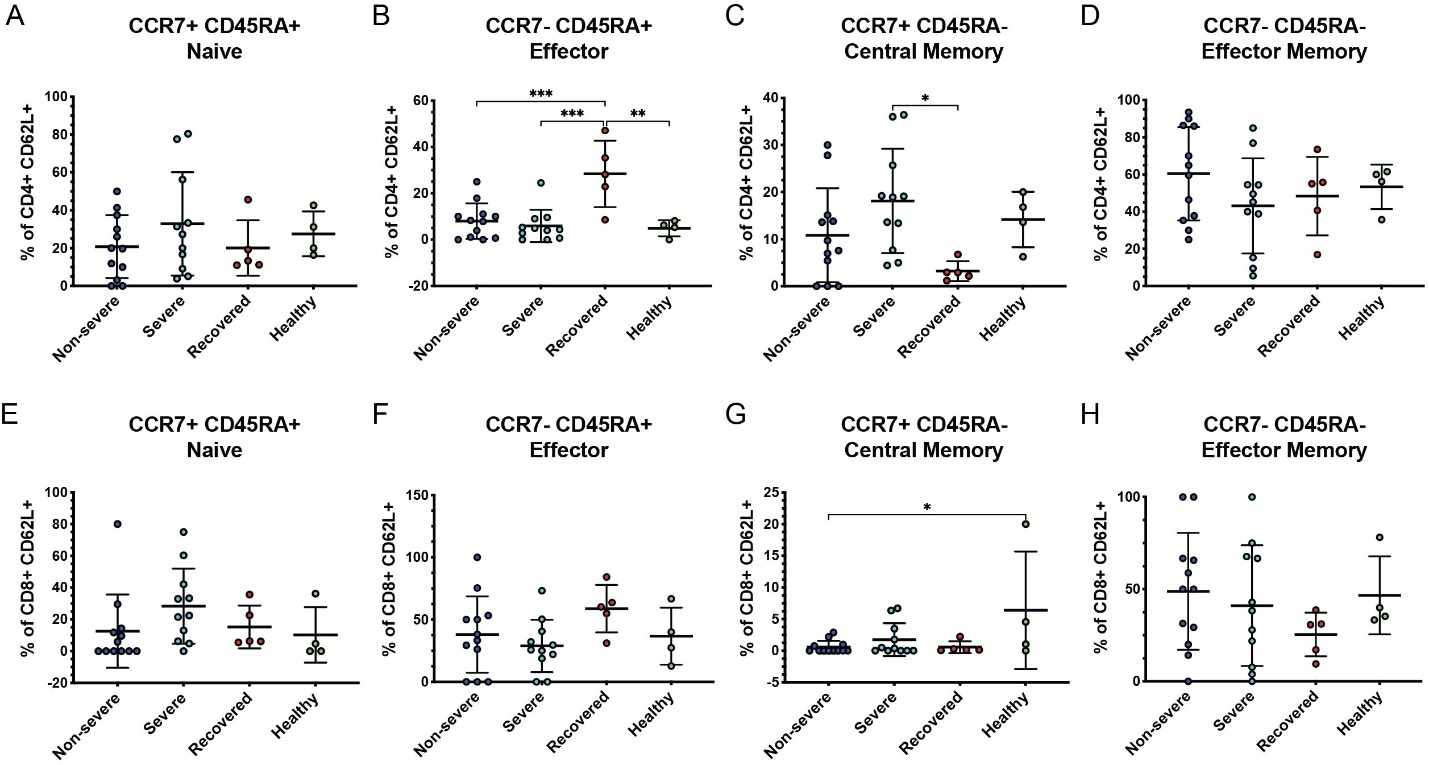 |
| --- |

**Figure S6. Expression of immunological memory markers on CD62L+ T cells.** Expression of CCR7 and CD45RA on CD62L+ CD4 and CD8 T cells in non-severe and severe COVID-19 patients, recovered COVID-19 patients, and healthy controls was assessed by spectral flow cytometry. (A) Percentage of naïve (CCR7+ CD45RA+), (B) effector/Temra (CCR7- CD45RA+), (C) central memory (CCR7+ CD45RA-), and (D) effector memory (CCR7- CD45RA-) cells within the CD4+ CD62L+ population; (E) Percentage of naïve, (F) effector/Temra, (G) central memory, and (H) effector memory cells within the CD8+ CD62L+ population. *p<0.05, **p<0.01, ***p<0.001 one-way ANOVA with multiple comparisons correction. Cells were gated on viable lymphocytes, CD14-, CD56-, CD19-, CD3+, CD4+ or CD8+ cells, CD62L+, and CCR7/CD45RA.

| 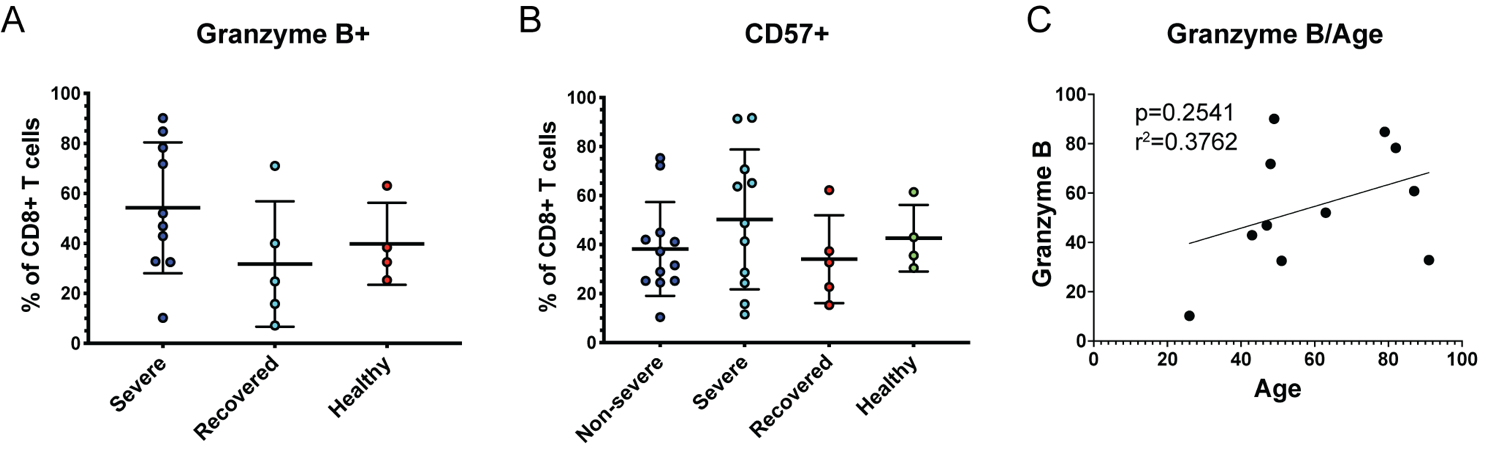 |
| --- |

**Figure S7. Granzyme B+ CD8+ T cells and CD57+ CD8+ T cells do not differ between acute COVID-19 patients, recovered patients, or healthy patients.** The frequencies of CD8+ T cells (viable, CD14-, CD56-, CD19-, CD3+, CD8+ cells) granzyme B (A) or CD57 (B) in non-severe and severe COVID-19 patients, recovered COVID-19 patients, and healthy controls were assessed by flow cytometry. C) Correlation between age and granzyme B+ CD8+ T cells.

**Supplemental Tables**

|  | **RBD and N Reactive** | **Only RBD Reactive** | **RBD and N Non-reactive** |
| --- | --- | --- | --- |
| **Malignancy or Immunocompromised (n=4)** | 2 | 1 | 1 |
| **HIV (n=1)** | 0 | 0 | 1 |
| **Diabetes (n=15)** | 10 | 4 | 1 |
| **Hypertension (n=8)** | 6 | 2 | 0 |
| **Coronary artery disease (n=5)** | 4 | 1 | 0 |
| **Pregnancy (n=1)** | 0 | 0 | 1 |

**Table S1. SARS-CoV-2 RBD and N ELISA reactivity in patients with comorbidities.**

| Reagent | Clone | Source | Catalog Number | Extracellular/  Intracellular |
| --- | --- | --- | --- | --- |
| Live/Dead Blue | N/A | Thermo Fisher | L34962 | Extracellular |
| CD8 BUV737 | RPA-T8 | BD Biosciences | 749367 | Extracellular |
| CD45RA BUV805 | HI100 | BD Biosciences | 742020 | Extracellular |
| Tim-3 BV480 | 3DS223H | BD Biosciences | 746771 | Extracellular |
| CD19 BV510 | HIB19 | Biolegend | 302242 | Extracellular |
| CD27 BV605 | O323 | Biolegend | 302830 | Extracellular |
| CD14 BV650 | M5E2 | Biolegend | 301836 | Extracellular |
| CD56 BV650 | HCD56 | Biolegend | 318344 | Extracellular |
| CCR7 BV750 | G043H7 | Biolegend | 353254 | Extracellular |
| CD25 PE | 2A3 | BD Biosciences | 341009 | Extracellular |
| CD4 CF568 | C4/206 | Biotium | BNC680206 | Extracellular |
| CD62L PE/Dazzle 594 | DREG-56 | Biolegend | 304842 | Extracellular |
| PD-1 PE-Cy7 | EH12.2H7 | Biolegend | 329918 | Extracellular |
| CD57 APC | NK-1 | BD Biosciences | 560845 | Extracellular |
| CD127 AF700 | A019D5 | Biolegend | 351344 | Extracellular |
| CD3 BUV496 | UCHT1 | BD Biosciences | 612940 | Intracellular |
| CTLA-4 BV421 | BNI3 | Biolegend | 369606 | Intracellular |
| FoxP3 AF488 | 236A/E7 | eBioscience | 53-4777-42 | Intracellular |
| Perforin PE | B-D48 | Biolegend | 353304 | Intracellular |
| Granzyme B | PE-CF594 | BD Biosciences | 562462 | Intracellular |

**Table S2. Antibodies for flow cytometry.**
